# Hybrid multivariate pattern analysis combined with extreme learning machine for Alzheimer’s dementia diagnosis using multi-measure rs-fMRI spatial patterns

**DOI:** 10.1371/journal.pone.0212582

**Published:** 2019-02-22

**Authors:** Duc Thanh Nguyen, Seungjun Ryu, Muhammad Naveed Iqbal Qureshi, Min Choi, Kun Ho Lee, Boreom Lee

**Affiliations:** 1 Department of Biomedical Science and Engineering (BMSE), Institute of Integrated Technology (IIT), Gwangju Institute of Science and Technology (GIST), Gwangju, Republic of Korea; 2 Translational Neuroimaging Laboratory, The McGill University Research Center for Studies in Aging (MCSA), McGill University, Montreal, Canada; 3 Alzheimer’s Disease Research Unit, Douglas Mental Health University Institute, McGill University, Montreal, Canada; 4 Department of Psychiatry, McGill University, Montreal, Canada; 5 Montreal Neurological Institute and Hospital, Montreal, Canada; 6 National Research Center for Dementia, Chosun University, Gwangju, Republic of Korea; 7 Department of Biomedical Science, Chosun University, Gwangju, Republic of Korea; Indiana University Purdue University at Indianapolis, UNITED STATES

## Abstract

**Background:**

Early diagnosis of Alzheimer’s disease (AD) and Mild Cognitive Impairment (MCI) is essential for timely treatment. Machine learning and multivariate pattern analysis (MVPA) for the diagnosis of brain disorders are explicitly attracting attention in the neuroimaging community. In this paper, we propose a voxel-wise discriminative framework applied to multi-measure resting-state fMRI (rs-fMRI) that integrates hybrid MVPA and extreme learning machine (ELM) for the automated discrimination of AD and MCI from the cognitive normal (CN) state.

**Materials and methods:**

We used two rs-fMRI cohorts: the public Alzheimer’s disease Neuroimaging Initiative database (ADNI2) and an in-house Alzheimer’s disease cohort from South Korea, both including individuals with AD, MCI, and normal controls. After extracting three-dimensional (3-D) patterns measuring regional coherence and functional connectivity during the resting state, we performed univariate statistical t-tests to generate a 3-D mask that retained only voxels showing significant changes. Given the initial univariate features, to enhance discriminative patterns, we implemented MVPA feature reduction using support vector machine-recursive feature elimination (SVM-RFE), and least absolute shrinkage and selection operator (LASSO), in combination with the univariate t-test. Classifications were performed by an ELM, and its efficiency was compared to linear and nonlinear (radial basis function) SVMs.

**Results:**

The maximal accuracies achieved by the method in the ADNI2 cohort were 98.86% (p<0.001) and 98.57% (p<0.001) for AD and MCI vs. CN, respectively. In the in-house cohort, the same accuracies were 98.70% (p<0.001) and 94.16% (p<0.001).

**Conclusion:**

From a clinical perspective, combining extreme learning machine and hybrid MVPA applied on concatenations of multiple rs-fMRI biomarkers can potentially assist the clinicians in AD and MCI diagnosis.

## Introduction

Alzheimer’s disease (AD) is the most common neurodegenerative disease and is the main cause of 60% to 70% of dementia cases in aging societies. It is characterized by cognitive decline and short-term memory loss [1, 2]. Mild cognitive impairment (MCI) is referred to as the prodromal stage of AD, and subjects with MCI are at high risk of developing AD [3]. Because AD/MCI are neurodegenerative diseases and progressively attack memory cells, the development of early diagnostic tools is undoubtedly important.

In recent years, resting-state functional magnetic resonance imaging (rs-fMRI) was shown to be a powerful tool for analysing the spontaneous blood-oxygen-level-dependent (BOLD) contrasts to map neural activity associated with a variety of brain functions. In order to map the brain areas involved in a given cognitive function, the BOLD signal at the level of the individual voxel is analyzed [4]. Statistical analysis is then performed on all voxels to show regions whose BOLD signal shows significant effects. This approach is referred to as univariate t-test analysis, which is performed independently on each voxel, and has been used in neuroimaging research for decades [5, 6]. However, this approach can only show differences between group averages, and is not sufficient to diagnose individual subjects. Therefore, recently, a machine learning (ML) technique known as multivariate pattern analysis (MVPA) has been promisingly applied to classify individual subjects using neuroimaging scans [7, 8]. Multivariate methods such as support vector machine-recursive feature elimination (SVM-RFE) and least absolute shrinkage and selection operator (LASSO) investigate the mutual relationships between multiple voxels and spatial patterns. Thus, the combination of univariate t-test and multivariate MVPA approaches is expected to enhance the prediction performance as compared to each individual approach used alone.

Previous fMRI studies have indicated that the pathophysiology of AD/MCI can be associated with statistical changes, in the average sense, of regional spontaneous low-frequency (<0.08 Hz) BOLD fluctuation coherence measured in the resting state and analysed using univariate t-tests. The metrics used in these studies included regional homogeneity (ReHo) [9, 10], amplitude of low-frequency fluctuation (ALFF) [11–13], and fractional ALFF (fALFF), as well as functional connectivity (FC) [14]. For example, He et. al., [10] showed that the posterior cingulate cortex (PCC) and the precuneus (PCu) have the largest ReHo differences between the AD and CN groups (p<0.05). The ALFF and fALFF studies using fMRI by Han et al., [15] revealed that MCI patients had decreased fALFF values in PCC/PCu and hippocampus, and increased fALFF values in several other regions, including occipital and temporal cortices. Rs-fMRI FC, investigated by Li et al. [16], showed that the regions with high FC were mostly located in the default mode network (DMN), and mainly involved the bilateral PCu and PCC [17]. These are all statistically significant findings at the group level. However, the discriminative ability based on the above-mentioned biomarkers related to AD/MCI diseases has not been evaluated. Since the discrimination task automatically classifies each subject into one of the studied groups (AD/MCI vs. CN), it is considered a much more complex task than the study of differences between groups [18, 19].

In neuroimaging studies, preprocessed brain scans commonly contain hundreds of thousands of non-zero voxels which significantly outnumber the number of subjects (often less than 1000). Thus, selection of an adequate subset of relevant training features/voxels is of critical importance to obtain good generalization ability and reduce risks of overfitting problems and computational complexity. A growing trend today is the design of ML-based feature reduction techniques integrated with classification methods applied to neuroimaging data for the voxel-based automated discrimination of patients with brain disorders, including AD and MCI (see the reviews [18, 19]). Many studies demonstrated the relevance of feature selection. Statistical hypothesis t-tests have broadly been used not only for group-discrimination detection but also for feature selections with success. The technique relies on an optimal threshold of significance (p-value) representing a subset of important features from whole-brain features. Though, applications of t-tests in feature selection are computational efficiency and easy to implement, this technique suffers from a significant drawback by not considering interactions between multiple features or spatial patterns which are the inherent multivariate nature of fMRI data. By contrast, MVPA methods do evaluate the relationships between multiple patterns. However, the primary drawback of whole-brain MVPA is its computationally demanding because of 3-D and high dimensionality of the data as well as the large number of images being analyzed [20–22]. Thus, to select the most informative features, a univariate feature selection strategy should be performed prior to MVPA in order to reduce the dimensionality sufficient for memory capacity, computational efficiency and ensure high sensitivity to fine-grained spatial discriminative patterns, while preserving the appealing properties of whole-brain fMRI analysis and multivariate nature of fMRI data [21, 22]. Practically, many previous studies have employed hybrid combinations of filter-based t-test and MVPA techniques, i.e. wrapper-based SVM-RFE, to diagnoze the brain disorders using neuroimaging data, e.g., ADHD [23–25], MCI [26–28], Autism [29], AD [30, 31], or for high-dimensional gene selections [22, 32] with success (accuracies>90%).

In this study, we propose a ML-based AD/MCI diagnosis framework combining MVPA and extreme learning machines (ELM) applied to multi-measure rs-fMRI data. We first extracted maps of 3-D regional coherence (ReHo, ALFF, and fALFF) and of resting-state FC (rsFC) (degree centrality (DC), seed-based rsFC) of multiple individual subjects. We then performed statistical univariate two-sample t-tests on whole-brain 3-D maps between two pre-defined training groups, to generate an analysis mask that retained only an initial set of relevant features (voxels) showing significant changes in any one of the measures, i.e. ReHo, fALFF, rs-FC. Next, MVPA techniques such as the wrapper-based SVM-RFE proposed by Guyon [20] and embedded-based LASSO were implemented to optimize the discriminative performance. In this study we used ELM and competing methods, including linear and non-linear SVM classifiers, to distinguish AD/MCI patients from the CN controls. We hypothesized that a hybrid combination of univariate statistical t-test and MVPA approaches applied on concatenation of multiple functional biomarkers could boost the classification performance. Thus, the major contributions present in this study can be summarized as follows:

- We propose a voxel-wise ML-based discriminative framework integrating ELM classifier and hybrid MVPA techniques for automated AD/MCI diagnosis using multi-measure rs-fMRI.
- The proposed framework extracts a maximum amount of information from multiple rs-fMRI biomarkers of a public Alzheimer’s disease Neuroimaging Initiative (ADNI2) and an in-house AD cohort from South Korea and, therefore, achieves maximal classification accuracies as compared to all other previous studies.
- We demonstrate that, compared to conventional univariate statistical analysis t-test, the hybrid combination of multivariate methods (univariate t-test + SVM-RFE and univariate t-test + LASSO) increases the classification performance of the discriminative patterns.
- The effectiveness of the ELM classifier, superior to that of linear and radial basis function (RBF)-based SVM classifiers, when combined with hybrid feature selection methods for AD/MCI identifications based on multi-biomarker rs-fMRI is addressed for the first time in this work.
- We showed that the highest classification accuracies are achieved when all patterns from multiple regional coherence and functional connectivity biomarkers are concatenated. This suggests that different brain regions suffer different functional losses due to AD/MCI. Hence, classification framework should include the maximum amount of informative changes to achieve best performance.

The remainder of this paper is organized as follows. Section 2 provides details on the datasets, subjects, preprocessing of rs-fMRI data, classification algorithms, univariate and MVPA feature reduction techniques, and permutation test used for the validation of the results. Section 3 presents the comparative results, while Section 4 is devoted to the discussion and conclusions of the article.

## Materials and methods

We used two independent rs-fMRI datasets: the ADNI2 dataset, publicly available online and an in-house dataset whose subjects were recruited from the Chosun University Hospital in Gwangju, South Korea.

### Subjects

#### ADNI2 cohort

We used a cohort of 33 (17 females) Alzheimer’s disease (AD) subjects, 31 (14 females) early Mild Cognitive Impairment (MCI) and 31 (17 females) Cognitive Normal (CN) subjects from the ADNI2 database, which is publicly available on the web (www.adni.loni.usc.edu). The mean ages of AD, MCI, and CN are 73.59 ± 5.18, 74.52 ± 5.18, and 74.66 ± 5.56. General criteria for categorizing AD, MCI, and CN are well explained on the ADNI web site (http://adni.loni.ucla.edu). The subjects ranged in age from 56 to 89 years, and functional assessments of AD/MCI patients, such as Mini-Mental State Examination (MMSE) and Clinical Dementia Rating (CDR), were independently performed by the research institutions. The general criteria were as follows: the CN subjects had MMSE scores between 24 and 30, a CDR of 0, and were non-depressed, non-MCI, and non-demented. MCI patients had MMSE scores between 24 and 30, CDR scores between 0 and 1, no significant levels of impairment in other cognitive domains, essentially preserved daily living activities, and absence of dementia. The MMSE scores of AD patients were between 15 and 26, their CDR scores were 0.5 or 1, and they met the National Institute of Neurological and Communicative Disorders and Stroke and the Alzheimer’s disease and Related Disorders Association (NINCDS/ADRDA) criteria for probable AD. In this study, to minimize the effect of different image sizes and resolutions, we selected images from subjects with the same image dimension and resolution, and we used only the baseline fMRI scans.

#### In-house cohort

A total of 365 subjects were included the in-house dataset: 81 AD subjects, 132 MCI subjects, and 152 CN subjects. This dataset was a part of a large cohort enrolled at the National Dementia Research Center, Chosun University, Gwangju, South Korea. All subjects provided written informed consent before the data collection. In case of AD patients with the inability of consent, the next of kin of patients gave consent before participation. Psychological tests or assessments were not used to determine whether subjects were able to provide written informed consent. The consent procedure and data acquisition were approved by the Institutional Review Board (IRB) of the Chosun University Hospital, Gwangju, South Korea (IRB number 2013-12-018). Briefly, subjects were between 56 and 87 years of age, and the study partners were able to provide independent functional evaluations. The MMSE and CDR scores, and the other clinical criteria for inclusion in the three groups were the same as in the ADNI2 cohort. The demographics of the participants from two cohorts are shown in Table 1 and subject IDs are provided in supporting S1 Table.

**Table 1:** Demographic details of all participants of two cohorts in this study.

**ADNI2 cohort**	**CN (*N = 31*)**	**MCI (*N = 31*)**	**AD (*N = 33*)**
Female/Male	17/14	14/17	17/16
Age years (Mean±STD)	74.66±5.56 (65–86)	74.52±4.97 (68–89)	73.59±5.18 (56–88)
MMSE score (Mean±STD)	29.29±1.48 (24–30)	27.67±1.8 (24–30)	21.54±3.32 (15–26)
CDR score (Mean±STD)	0±0 (0–0)	0.50±0.17 (0–0.5)	0.89±0.21 (0.5–1)
**In-house cohort**	**CN (*N = 152*)**	**MCI (*N = 132*)**	**AD (*N = 81*)**
Female/Male	79/73	64/68	42/39
Age years (Mean±STD)	71.44±5.47 (60–85)	73.10±5.92 (59–87)	71.86±7.09 (56–83)
MMSE score (Mean±STD)	28.29±1.07 (24–30)	26.83±2.49 (24–30)	18.56±1.95 (14–24)
CDR score (Mean±STD)	0±0 (0–0)	0.47±0.36 (0–0.5)	0.91±0.26 (0.5–1)

Abbreviation: MMSE: Mini-Mental State Examination. CDR: Clinical Dementia Rating. N = number of subjects. STD: standard deviation.

### Rs-fMRI data acquisition

#### ADNI2 cohort

ADNI2 subjects were scanned at different centres using 3.0 T Philips Achieva scanners with the same scanning protocol and parameters: Repetition Time (TR)/Echo Time (TE) = 3000/30 ms, flip angle = 80°, acquisition matrix size = 64 × 64, 48 slices, 140 volumes, and a voxel thickness = 3.3 mm.

#### In-house cohort

The participants in the Chosun University Hospital were scanned with a Siemens Skyra 3.0-Tesla scanner. A 2D EPI MR acquisition type was used with the following parameters: TR/TE = 3000/30 ms, flip angle = 90°, field of view (FOV) = 240 × 240 mm, acquisition matrix size 64 × 64, 35 slices, 90 volumes, voxel size = 3.75 x 3.75 x 3.75, spacing between slices = 4.8 mm, number of echoes = 1, imaging frequency = 123.206 Hz, slice acquisition order = ascending (bottom-up), direction = 'Transverse > Coronal (2.6) > Sagittal (1.7)', pixel bandwidth = 3440, in-plane phase encoding direction = ‘ROW’, number of phase encoding steps = 63, echo train length = 31, percent sampling = 100, percent phase field of view = 100, variable flip angle flag = ‘N’, and specific absorption rate (SAR) = 0.0778.

### Preprocessing of rs-fMRI data

Preprocessing of rs-fMRI data was carried out using the Data Processing Assistant for Resting-State fMRI (DPARSF; http://www.restfmri.net) [33] and the Statistical Parametric Mapping platform (SPM8; http://www.fil.ion.ucl.ac.uk/spm). All Digital Imaging and Communications in Medicine (DICOM) files were obtained from the scanners as described above, and converted into the Neuroimaging informatics Technology initiative (NIfTI) file format. The first 10 time points for each participant were disregarded to allow for signal calibration and participants’ adaption to the scanning noise. Subsequently, functional images went through the following preprocessing steps: slice-timing correction was referred to the last slice; realignment for head movement compensation was performed by applying a Friston 24-parameter model (6 head motion parameters, 6 head motion parameters from the previous time point, and 12 corresponding squared items); individual structural images (T1-weighted MPRAGE) were co-registered to the mean functional image after realignment; normalization the rs-fMRI to the original space was performed with the Diffeomorphic Anatomical Registration Through Exponentiated Lie algebra (DARTEL) toolbox [34] (resampling voxel size = 3 × 3 × 3 mm^3^); spatial smoothing was performed with a 6-mm full-width at half-maximum (FWHM) Gaussian kernel. Then, linear trend removal and temporal band-pass filtering (0.01 Hz < f < 0.08 Hz) were performed on the time series of each voxel. Finally, we regressed out cerebrospinal and white matter signals as well as six head-motion parameters to further reduce the effects of nuisance signals and focus only on the gray matters signal. A mask image was created according to the intersection of the subject-specific normalized T1 anatomical images. Only the voxels within the mask were further analyzed. The mask image was also used for correcting for multiple comparisons in later analyses.

### Proposed framework

**Fig 1:**
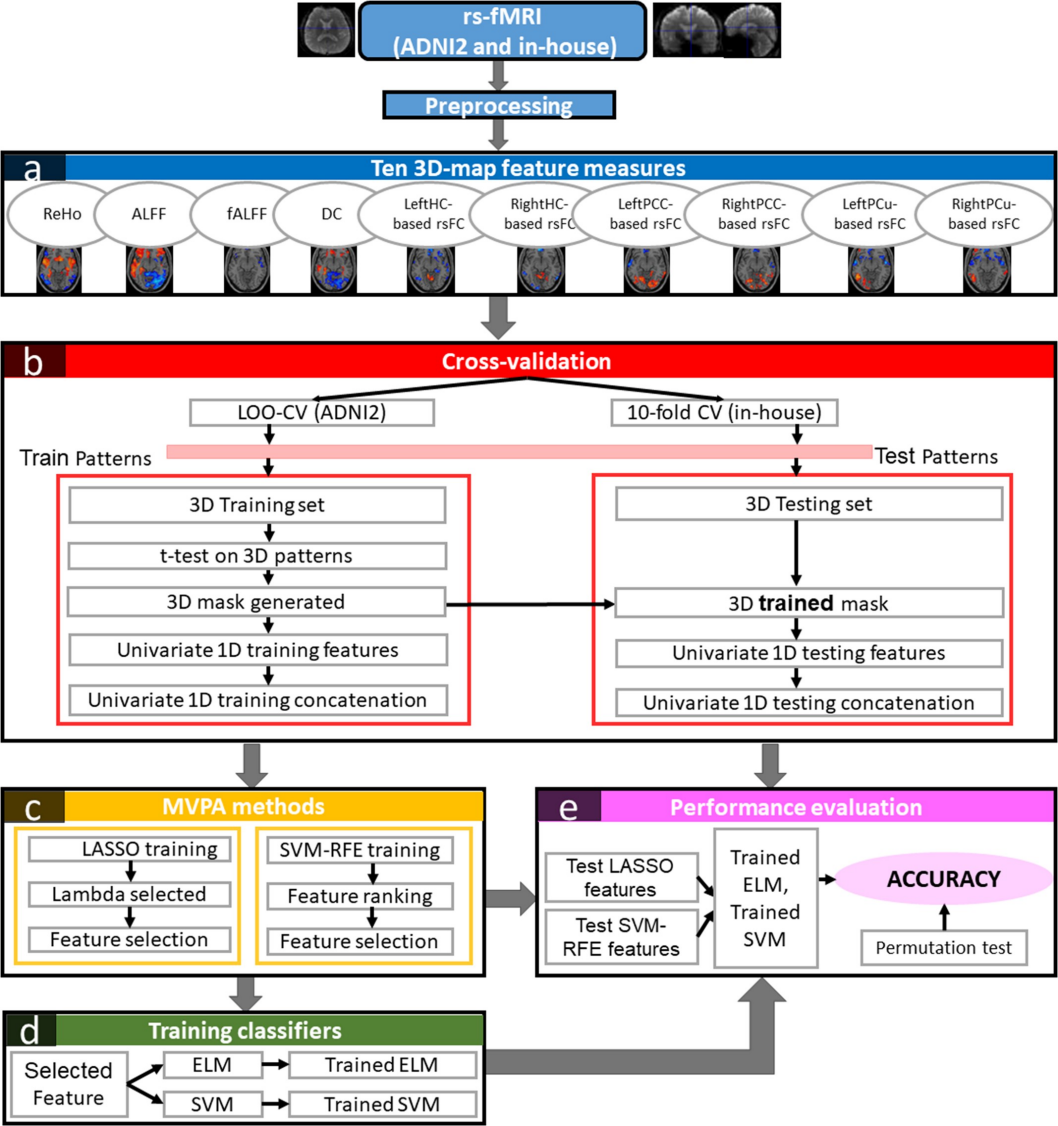
Descriptions of the proposed framework in this study. Block (a) presents the 3-D feature measure extractions from preprocessed fMRI scans. Block (b) describes the LOO-CV and 10-fold-CV cross validation for ADNI2 and in-house cohorts, respectively. Block (c) presents the multivariate feature reduction techniques using LASSO and SVM-RFE. The combined univariate t-test and multivariate LASSO as well as SVM-RFE informative features are trained by ELM and SVM classifiers as illustrated in block (d). Finally, the trained classifiers and testing features are used to evaluate the performance as in block (e).

Fig 1 illustrates all the procedures and techniques proposed in this study. The first step of the procedure is to extract the whole-brain 3-D measures from processed rs-fMRI images. The measures are ReHo, fALFF, ALFF, Degree Centrality (DC), left hippocampus-based rsFC (LeftHC-based rsFC), right hippocampus-based rsFC (RightHC-based rsFC), left post cingulate cortex-based rsFC (LeftPCC-based rsFC), right post cingulate cortex-based rsFC (RightPCC-based rsFC), left precuneus-based rsFC (LeftPCu-based rsFC), and right precuneus-based rsFC (RightPCu-based rsFC). Due to the small size of the datasets, we used leave-one-out (LOO-CV) and 10-fold cross-validation (10-fold CV) for the ADNI2 and the in-house cohort, respectively, to validate the classification performance of the methods. In LOO-CV, one sample was selected as testing data whereas the rest was used for training. In 10-fold CV, 90% of the data were used for training and the remaining 10% for testing. Given training 3-D spatial maps, we then performed univariate statistical t-tests to obtain a 3-D mask which identified a set of ‘active’ voxels. We then implemented the MVPA techniques (SVM-RFE and LASSO) on 1-D concatenated training features to select the most relevant features for training the ELM and SVM classifiers. Finally, given the indices of the highest ranked features on the training data, we extracted the testing data for classification.

### Feature extraction

We describe here some biomarkers measured from rs-fMRI using the Resting-State fMRI Data Analysis Toolkit (REST) toolbox [35]. The measures can be categorized into regional spontaneous measures (ReHo, ALFF, fALFF), and functional connectivity measures (DC, seed-based rsFC), as described below.

#### Regional homogeneity (ReHo)

We used the ReHo measure to explore regional brain activity during the resting state. The computation was performed on a voxel-wise basis by calculating Kendall’s Coefficient of Concordance (KCC) [36] of fMRI time series of a given voxel with those of its nearest neighbours. From all the voxels in the brain, an individual ReHo map was obtained for each subject. A higher regional coherence within a cluster, consisting of a voxel and its nearest neighbours, was represented by a larger ReHo value for the voxel. Several recent studies in literature have shown the potential value of ReHo in clinical applications [9, 10, 37].

#### Amplitude of low-frequency fluctuation (ALFF) and fractional ALFF (fALFF)

The regional spontaneous activities can be examined by the ALFF measure and its improved version, the fALFF measure. After preprocessing, the filtered time series was transformed to a frequency domain using a fast Fourier transform (FFT), and the power spectrum was obtained. The average square root of the power spectrum (amplitude) between the frequencies of 0.01 and 0.08 Hz was computed at each voxel to give the ALFF measure [11, 38]. The fALFF measure is a modified version of ALFF, defined as the ratio of the average amplitude in the low-frequency range (0.01–0.08 Hz) to that of the entire frequency range (0–0.25 Hz) [33].

#### Degree centrality (DC)

We used a commonly employed graph-based measure of network organization, degree centrality (DC), to perform a full-brain exploration of the regions that were influenced by AD and MCI. Within the study mask, individual network centrality maps were generated in a voxel-wise fashion. First, the preprocessed functional runs were subjected to voxel-based whole-brain correlation analysis. The time course of each voxel from each participant that was within the gray matter mask was correlated with the time course of every other voxel, to obtain a correlation matrix. An undirected adjacency matrix was then obtained by thresholding the correlation at r > 0.25 [39, 40], and the DC was computed as the sum of the weights of the significant weighted connections for each voxel. Finally, the individual-level voxel-wise DC was converted into a z-score map by subtracting the mean DC across the entire brain and dividing by the standard deviation of the whole-brain DC.

#### Seed-based resting-state functional connectivity (rsFC)

To examine the detailed rsFC differences among the AD, MCI and CN groups at the regional level, we performed seed-based rsFC analysis. Briefly, the mean time course within each seed was extracted by averaging the time courses of all the voxels belonging to the seed. Subsequently, the mean time course was used to compute the correlation coefficients with the time courses of all voxels. The resulting correlation coefficients were then converted to z-scores using Fisher’s r-to-z transform to improve normality [16, 41]. In this study, we selected bilateral PCC, bilateral Hippocampus, and bilateral Precuneus as the seeds. Table 2 provides detailed information about the seeds.

**Table 2:** Detailed information of the seeds for seed-based rsFC measures.

Seed	Abbreviation in this study for seed-based rsFC	ROI in 116-ROI AAL	Coordination, mm		
			x	y	z
Left Post Cingulate Cortex (LeftPCC)	LeftPCC-based rsFC	35	-6	-43	25
Right Post Cingulate Cortex (RightPCC)	RightPCC-based rsFC	36	6	-42	22
Left Hippocampus (LeftHC)	LeftHC-based rsFC	37	-26	-21	-10
Right Hippocampus (RightHC)	RightHC-based rsFC	38	28	106	62
Left Precuneus (LeftPCu)	LeftPCu-based rsFC	67	-8	-56	48
Right Precuneus (RightPCu)	RightPCu-based rsFC	68	9	-56	44

#### Feature concatenation

Combining multiple measures is a very effective approach for boosting the performance of a machine learning setup [42], which has been used in many research domains, including neuroimaging classification [43]. In this work, we investigated a common feature concatenation that linked many feature measures of the same dataset. We believe that feature concatenation will enhance accuracy and enable the inference of indirect or direct associations between multiple features extracted from the same fMRI data.

### Feature reduction techniques

The number of predictor voxels obtained in our spatial maps was larger than the number of subjects. Thus, a dimensionality reduction process was necessary in order to select the most relevant features, discard redundant features and noise, and avoid numerical singularities and overfitting problems, and thus enhance the classification performance. Importantly, feature reduction was performed using the training data only. Once identified, the same brain regions identified during training were used to assess the classifier predictive accuracy [44] on the testing data. In this study, we used univariate t-test and MVPA approaches, including SVM-RFE and LASSO, as voxel-wise feature reduction techniques. The univariate t-test is performed voxel-wise to identify independent voxels, whereas the multivariate RFE and LASSO investigate the mutual associations between multiple features and spatial patterns. We also used hybrid combinations of univariate and MVPA approaches to outperform the individual techniques.

#### Univariate two sample t-test

**Fig 2:**
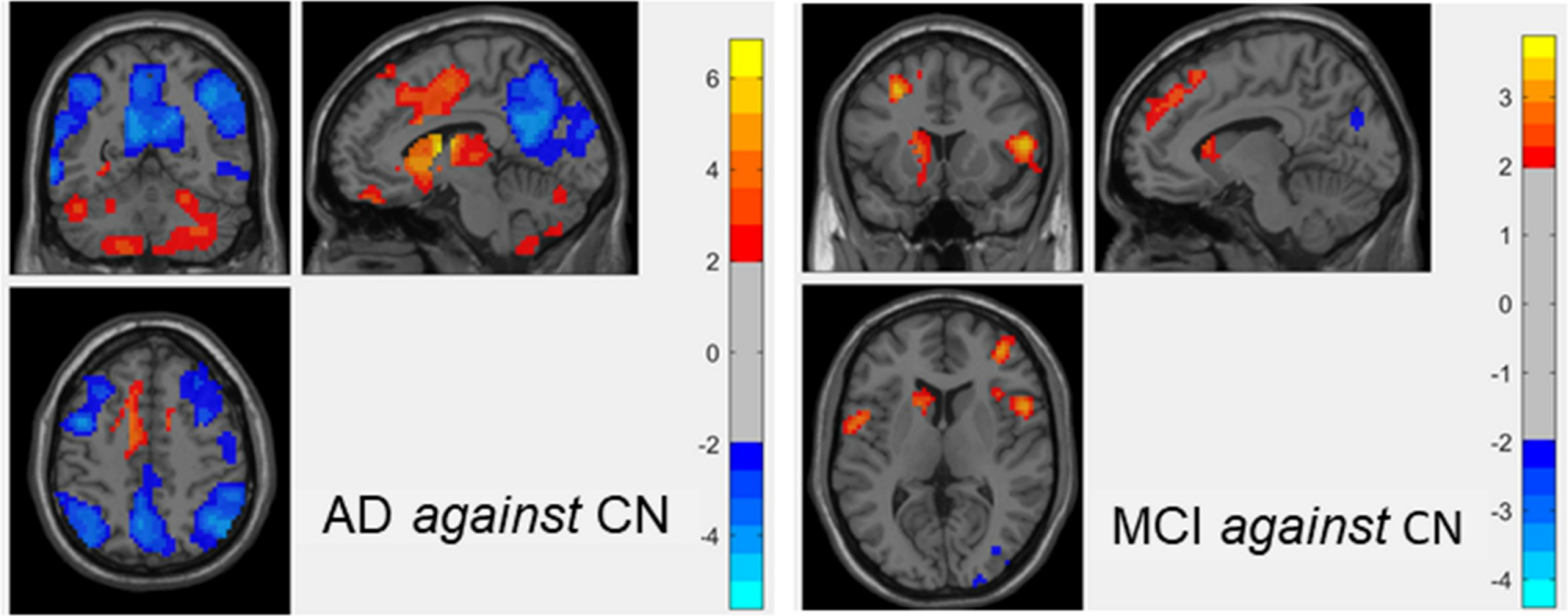
**An example of one-fold univariate statistical two-sample t-test on ReHo maps between two training analytical groups, i.e., AD against CN (left subfigure) and MCI against CN (right subfigure).** The threshold was set to p-value<0.05 with cluster size of 85 voxels (2295 mm^3^), which corresponded to a corrected p-value<0.05. The t-test maps are overlaid on the anatomical image. The hot and cold colours represent positive and negative changes.

Many neuroimaging studies have shown abnormalities, at the level of the average signal, in one or more brain features in a diseased group compared to a control group using univariate statistical tests [19]. Recently, classification studies have used t-tests to select informative features for machine learning in neuroimaging [8, 45]. The key results of the analysis based on statistical tests are usually expressed by means of p-values. Subsequently, the optimal p-value cutoff to select the relevant features is determined through a cross-validation process, and the features thus selected are used in the subsequent machine learning analysis. In this study, we applied t-test-based feature reductions techniques to machine learning based diagnosis. Using t-tests on the training dataset, we generated an analytical mask that retained only the voxels presenting significant changes in any of the analytical feature measures, i.e. ReHo, ALFF, fALFF, DC, rsFC, between any of the two groups at the threshold p-values (p<0.05 with |t|>1.9715, p<0.01 with |t|> 2.599, and p<0.001 with |t|>3.3381). The correction cluster size threshold p = 0.05 corresponding to corrected individual voxel p-values was computed by Monte Carlo simulations with the program AlphaSim in REST [35] (1000 iterations) to determine the cluster size. As a result, cluster sizes of 85 voxels (2295 mm^3^), 18 voxels (486 mm^3^), 6 voxels (162 mm^3^) were found to correspond to corrected individual voxel p-values of 0.05, 0.01, and 0.001, respectively. Fig 2 shows selected regions resulted from univariate t-test applied to ReHo maps of one-fold training data, i.e., AD vs. CN and MCI vs. CN (out of >62 different folds for ADNI2 cohort and 10 folds for in-house cohort).

#### Support vector machine-recursive feature elimination (SVM-RFE)

**Fig 3:**
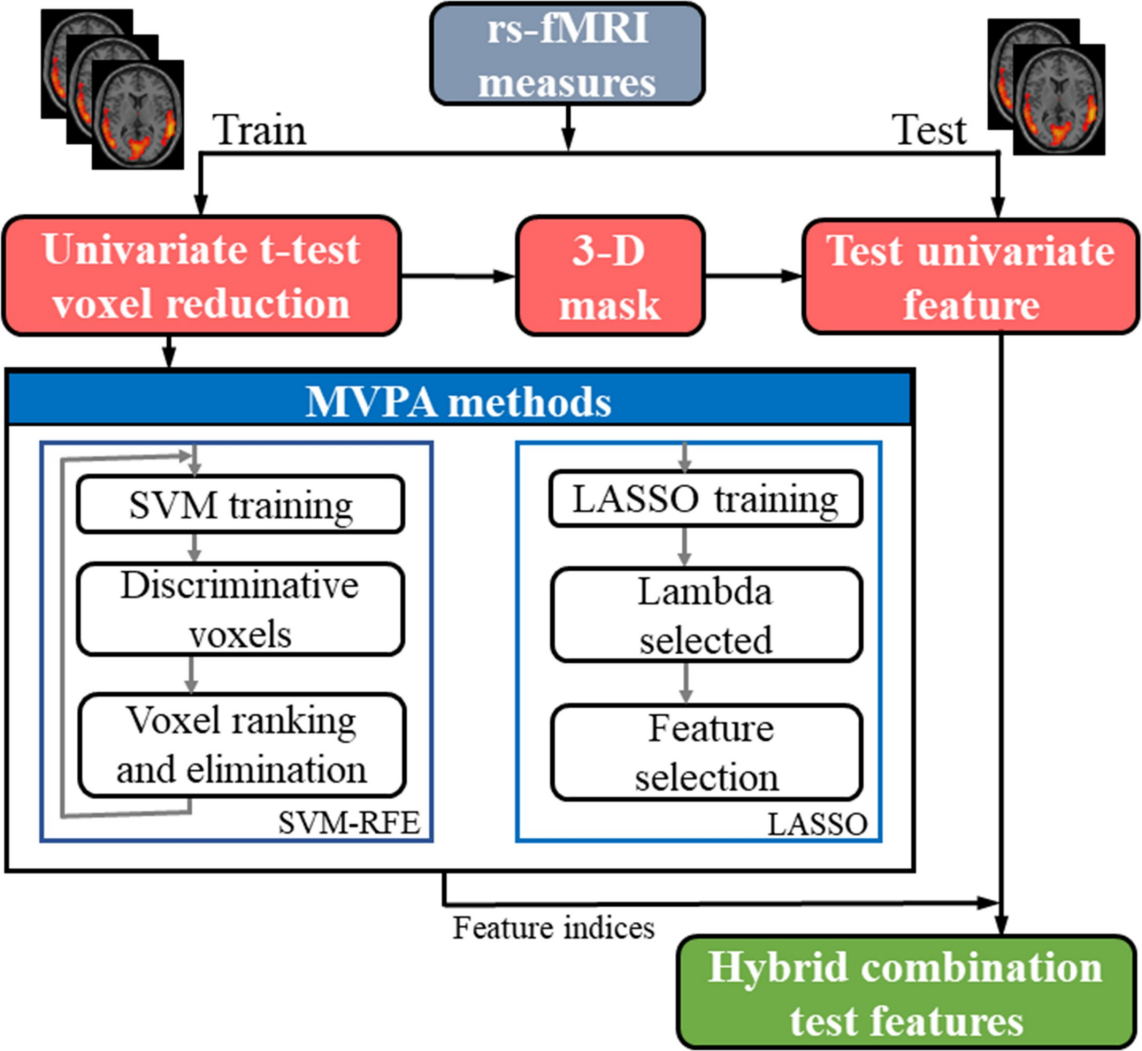
Illustration of the hybrid combination of univariate t-test and MVPA feature reduction techniques (SVM-RFE and LASSO) on the 3-D cross-validated fMRI measures.

While the t-test is a univariate procedure that does not take into account interactions between multiple features and spatial patterns, support vector machine-recursive feature elimination (SVM-RFE) is a multivariate wrapper-model-based feature reduction algorithm, which efficiently fits a model and removes the weakest features until the specified informative number of features is reached. The ranking criterion of SVM-RFE is closely related to the SVM model. In each iteration of the RFE, an SVM model is trained. Then, the feature with smallest ranking criterion is removed since it has the least effect on classification, while the remaining features are kept for the SVM model in the next iteration. The sequential process is repeated until all the features have been eliminated. Then, according to the order of elimination, the features are graded. The later a feature is eliminated, the more significant it should be [46]. A detailed description of the SVM-RFE algorithm can be found in a previous paper [20]. In this work, after the application of SVM-RFE, the most important training features that maximize cross-validated accuracy were kept for training the classifiers. Fig 3 illustrates the process of hybrid combination of univariate t-test and multivariate SVM-RFE as well as LASSO to select the most relevant features.

#### Least absolute shrinkage and selection operator (LASSO)

**Fig 4:**
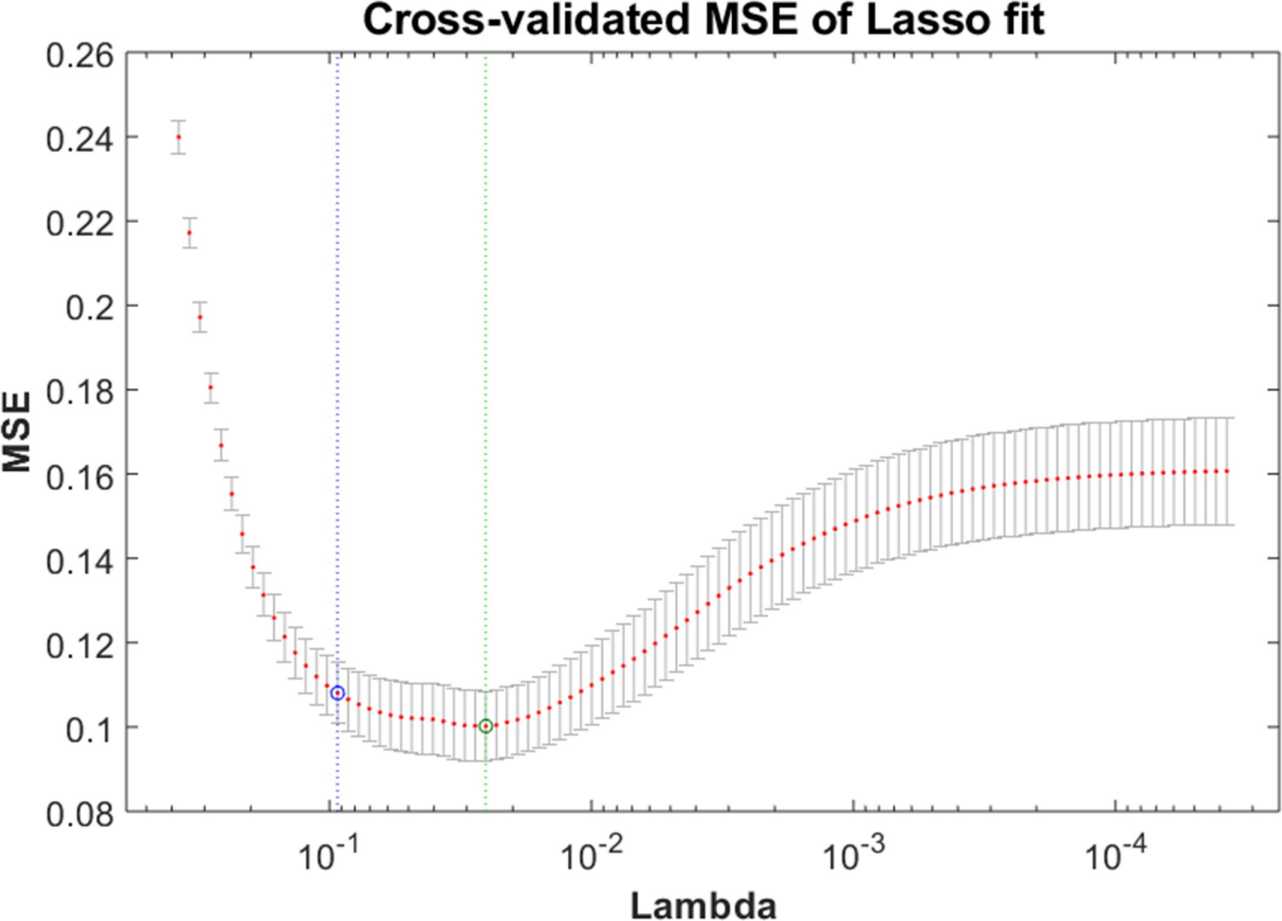
An example of cross-validated MSE of LASSO fit with a parameter lambda (λ).

A good example of MVPA feature reduction with error and regularization terms is LASSO, which has been successfully applied in neuroimaging machine learning tasks to mitigate problems related to the so-called *curse-of-dimensionality*. LASSO computes model coefficients *γ_j_* by minimizing the following function:

$$\underset{\gamma_{0}\gamma_{j}}{\min}\left(\frac{1}{2n}\sum_{i=1}^{n}{\left(u_{i}-\gamma_{0}-x_{i}^{T}\gamma_{j}\right)}^{2}+\lambda \sum_{j=1}^{q}\left|\gamma_{j}\right|\right)$$

where *x_i_* is the voxel-wise feature input data, a vector of *q* values at observation *i*, and *n* is the number of observations. *u_i_* is the response at observation *i*. Lambda (λ) is a non-negative user-defined regularization parameter which controls the balance between limiting the number of non-zero coefficients *γ_j_* (sparsity) and high prediction accuracy. Interestingly, as λ approaches 1, the model becomes increasingly sparse, meaning it will produce few relevant features, while as λ approaches 0, the model becomes less sparse and includes more relevant features [5]. The parameter *γ*_0_ is a scalar. The function minimized by LASSO involves the *l*_1_ norm of *γ_j_* [47–49]. In this paper, we chose the value of λ that minimized the cross validated mean squared error (MSE), as shown in Fig 4. The hybrid combination of univariate t-test and multivariate LASSO for selecting the most discriminative training features is shown in Fig 3.

### Classification

In this study, three machine learning classification algorithms were used namely, ELM, linear SVM, and non-linear SVM. We have compared the results of all the classifiers, and ELM proves to be the most efficient algorithm both in terms of computation time and accuracy. Brief description of each method is described as follows.

#### ELM classifier

An ELM consists of an input layer, a hidden layer, and an output layer. Whereas traditional feedforward neural networks require weights and biases for all layers to be adjusted by gradient-based learning algorithms, ELM arbitrarily assigns input weights and hidden layer biases without iterative adjustment, and computes the output weights by solving a single linear system [23]. Thus, ELM learns much faster than traditional neural networks and is widely employed in various classification applications as an efficient learning algorithm [24]. In this work, the number of hidden nodes was set between 1 and 400, and we selected a sigmoid activation function. A grid search method on training data was used to tune this parameter for achieving maximum cross-validated validation accuracy. To minimize the random effects due to the weight initializations, each value of the number of hidden nodes was used 100 times and the average performance was presented.

#### SVM classifier

Support vector machines (SVM) have recently become popular as supervised classifiers of fMRI data due to their high performance, their ability to deal with large high-dimensional datasets, and their flexibility in modeling diverse sources of data [4]. In the present study, we utilized a linear SVM and a non-linear SVM based on radial basis function (RBF) kernels. In SVMs, the parameters that need to be tuned are the gamma value of the kernel scale (γ) and the box constraint (C). We used a greedy search method on training data to tune these parameters to maximize cross-validated test accuracy. In this study, the search scale for selecting gamma values of kernel scale and box constraint were set to γ = [0.001, 0.01, 0.1, 1, 10, 100, 1000, 10000], and C = [0.001, 0.01, 0.1, 1, 10, 100, 1000, 10000], respectively.

### Cross-validation, performance evaluation, and significant testing methods

#### Cross-validation

In this work, we used Leave-One-Out cross-validation (LOO-CV) for the ADNI2 cohort and 10-fold cross-validation (CV) for the in-house cohort. In the LOO-CV, N-1 subjects out of N were used for training, and the remaining one was left for testing, and the procedure was repeated for all the N subjects. In 10-fold CV, the subjects were randomly divided into 10 equally sized subsets: each of these subsets (folds), containing 10% of the subjects, was then used as testing set for a model trained on the remaining 90%. The mean performance of all N test subjects in LOO-CV, or all the 10 folds in 10-fold CV was reported as the final result.

#### Performance evaluation

To evaluate the performance of the classifiers, we reported accuracy (ACC), sensitivity (SEN), specificity (SPEC), balanced accuracy (BAC), positive predictive value (PPV), and negative predictive value (NPV). TP, TN, FP, and FN indicate the number of true positives, true negatives, false positives, and false negatives, respectively. In terms of these numbers, ACC, SEN, SPEC, BAC, PPV, and NPV can be computed as follows:

Accuracy (ACC) = (TP + TN)/(TP + TN + FP + FN)
Sensitivity (SEN) = TP/(TP + FN)
Specificity (SPEC) = TN/(TN + FP)
Balanced accuracy (BAC) = (SEN + SPEC)/2
Positive predictive value (PPV) = TP/(TP + FP)
Negative predictive value (NPV) = TN/(TN + FN)

#### Significant testing methods

To assess the statistical significance of the classifiers’ performance, a permutation test was performed on the classification accuracies, by randomly permuting 1000 times the labels of the test data of each of the N (LOO-CV) or 10 (10-fold CV) folds to get the probability of random successful classification. In general, the lower the p-value of the permuted prediction rate against the prediction rate of the original data labels, the higher the significance of the classifier performance.

## Results

### Classification results: Univariate t-test

#### ADNI2 cohort

Tables 3 and 4 summarize the classification performance in discriminating between AD and CN, and between MCI and CN, respectively, of all the competing methods on the ADNI2 cohort. In terms of the mean diagnosis accuracy, the ELM classifier with concatenated features obtained a maximal accuracy of 89.92% (p-value<0.001) with a sensitivity of 86.51%, specificity of 84.17%, balanced accuracy of 84.58%, PPV of 94.00%, and NPV of 87.40% when discriminating between AD and CN; and a maximal accuracy of 85.81% (p-value<0.001) with a sensitivity of 86.67%, specificity of 85.83%, balanced accuracy of 84.85%%, PPV of 86.50% and NPV of 90.00% when discriminating between MCI and CN. The concatenated measure outperformed all individual measures. In addition, the ELM outperformed the linear and RBF-based non-linear SVMs in terms of diagnosis accuracy in all measures, including concatenated ones.

**Table 3:** Leave-One-Out cross-validation mean classification performance for AD versus CN of multi-measure features at p-value = 0.05 with ADNI2 cohort.

Feature Measure	ELM								SVM-RBF	SVM-Linear
	ACC Training (%)	ACC Testing (%)	p-value	SEN Testing (%)	SPEC Testing (%)	BAC Testing (%)	PPV Testing (%)	NPV Testing (%)	ACC Testing (%)	ACC Testing (%)
ReHo	89.57±4.10	84.29±1.23	0.001	85.00±1.10	83.33±1.57	84.17±1.76	88.00±2.74	88.56±1.61	66.43±5.49	71.43±3.65
fALFF	83.16±2.18	85.07±2.34	0.001	95.00±5.41	73.33±4.05	84.17±1.76	84.00±8.34	95.00±10.45	71.90±3.91	75.00±3.05
ALFF	85.30±5.29	85.71±1.14	0.001	97.50±3.91	70.00±5.41	83.75±1.32	82.00±6.32	97.50±7.91	75.38±1.67	76.62±7.90
Degree Centrality	87.63±1.05	87.15±1.53	0.001	90.00±2.92	80.00±7.21	85.00±2.15	88.33±10.56	90.00±12.91	70.24±6.04	73.57±4.78
LeftHC-based rsFC	93.51±4.91	85.51±3.16	0.001	85.00±4.91	86.67±1.24	85.53±2.15	92.00±10.33	85.00±12.91	71.67±4.26	73.33±4.58
RightHC-based rsFC	96.49±4.14	87.75±1.63	0.001	80.00±5.54	93.33±4.05	86.67±1.76	96.00±8.43	80.00±10.54	59.52±1.35	65.71±2.15
LeftPCC-based rsFC	81.36±4.26	84.47±2.17	0.001	90.00±2.91	80.00±7.21	85.00±2.15	88.00±10.33	90.00±12.91	71.67±4.82	73.33±2.93
RightPCC-based rsFC	83.33±1.12	81.75±3.35	0.001	85.00±5.31	86.67±1.29	85.83±2.15	92.00±10.33	85.00±12.91	71.19±1.92	71.19±1.92
LeftPCu-based rsFC	94.04±6.76	83.49±2.08	0.001	82.50±2.04	90.00±6.10	86.25±2.01	94.00±6.96	82.50±12.43	64.05±3.11	70.48±6.66
RightPCu-based rsFC	87.42±4.90	85.71±3.64	0.001	87.50±13.18	83.33±3.62	85.42±2.22	90.00±5.40	87.50±3.18	71.90±2.18	70.71±5.87
**Concatenation**	**94.07**±**3.81**	**89.92**±**1.23**	**0.001**	**86.51**±**6.10**	**84.17**±**6.87**	**84.58**±**2.01**	**94.00**±**2.74**	**87.40**±**3.18**	**76.19**±**2.09**	**77.62**±**2.31**

Abbreviation: ReHo = regional homogeneity; ALFF = amplitude of low-frequency fluctuation; fALFF = fractional amplitude of low-frequency fluctuation; LeftHC-based rsFC = Left Hippocampus seed-based rsFC; RightHC-based rsFC = Right Hippocampus seed-based rsFC; LeftPCC-based rsFC = Left Post Cingulate Cortex seed-based rsFC; RightPCC-based rsFC = Right Post Cingulate Cortex seed-based rsFC; LeftPCu-based rsFC = Left Precuneus seed-based rsFC. RightPCu-based rsFC = Right Precuneus seed-based rsFC. The bold results indicate the maximal performances.

**Table 4:** Leave-One-Out cross-validation mean classification performance for MCI against CN of multi-functional features at p-value = 0.05 with ADNI2 cohort.

Feature Measure	ELM								SVM-RBF	SVM-Linear
	ACC Training (%)	ACC Testing (%)	p-value	SEN Testing (%)	SPEC Testing (%)	BAC Testing (%)	PPV Testing (%)	NPV Testing (%)	ACC Testing (%)	ACC Testing (%)
ReHo	91.55±2.70	85.01±3.25	0.001	88.50±6.32	80.00±7.12	84.75±1.32	85.00±2.91	90.50±2.35	70.95±1.53	72.71±4.10
fALFF	80.90±4.08	81.95±5.12	0.001	85.68±2.71	80.00±2.17	82.34±1.75	85.50±2.57	90.50±2.35	68.10±9.70	65.00±6.91
ALFF	79.55±4.10	84.65±3.06	0.001	90.83±4.93	76.67±6.10	83.75±1.32	82.50±2.08	93.00±1.35	72.62±2.81	73.95±3.93
Degree Centrality	88.80±4.80	82.13±1.94	0.001	85.07±4.21	80.83±6.69	82.85±1.32	85.50±2.57	90.00±2.91	70.95±1.44	70.62±5.02
LeftHC-based rsFC	83.58±5.22	82.04±4.26	0.001	87.00±6.10	77.50±5.74	82.25±1.32	83.00±1.83	92.50±2.08	70.95±1.04	65.00±4.98
RightHC-based rsFC	88.96±2.75	80.47±5.13	0.001	73.33±4.05	89.33±4.26	81.33±0.00	93.50±5.69	80.50±1.39	64.52±5.13	64.52±5.89
LeftPCC-based rsFC	88.27±7.63	84.02±3.35	0.001	77.50±5.74	90.83±4.93	84.17±1.76	92.50±2.08	82.50±1.84	67.62±3.15	60.95±7.97
RightPCC-based rsFC	86.60±5.11	81.14±2.87	0.001	84.67±5.15	80.83±6.69	82.75±1.32	85.50±2.57	90.00±2.91	70.71±4.11	69.05±6.84
LeftPCu-based rsFC	86.99±5.03	83.57±3.41	0.001	90.83±4.93	77.50±5.74	84.17±1.76	82.50±2.08	92.50±1.28	71.62±5.89	71.19±6.36
RightPCu-based rsFC	87.88±4.25	82.75±4.36	0.001	83.17±6.87	83.17±6.87	83.17±1.75	87.50±3.18	87.50±3.18	72.82±7.43	69.29±9.97
**Concatenation**	**87.54**±**2.77**	**85.81**±**3.53**	**0.001**	**86.67**±**2.71**	**85.83**±**6.69**	**84.85**±**1.32**	**86.50**±**2.57**	**90.00**±**2.91**	**76.62**±**5.07**	**77.69**±**5.26**

#### In-house cohort

The experimental results on the in-house dataset are summarized in Table 5 (AD against CN) and Table 6 (MCI against CN). Our proposed method with the ELM classifier achieved very high mean accuracies for all types of measures (above 90% mean accuracy for AC against CN; around 80% mean accuracy for MCI against CN). Note that, in AD vs. CN, the concatenation of all measures resulted in a maximal mean accuracy of 94.45% (p-value<0.001) with a sensitivity of 83.67%, a specificity of 96.67%, a balance accuracy of 90.17%, a PPV of 95.67%, and a NPV of 91.07%; in MCI vs. CN, the maximal mean accuracy was 87.20% (p-value<0.001), with a sensitivity of 78.85%, a specificity of 87.50%, a balance accuracy of 81.69%, a PPV of 84.66%, and a NPV of 81.27%. Again, the performance of the combined measures was superior to that of the individual measures. In addition, the mean accuracy of the ELM classifier was superior to that of the linear and non-linear SVMs, as can be seen in the Tables 5 (AD vs. CN) and 6 (MCI vs. CN).

**Table 5:** 10-fold cross-validation mean classification performance for AD against CN of multi-functional features at p-value = 0.05 with In-house cohort.

Feature Measure	ELM								SVM-RBF	SVM-Linear
	ACC Training (%)	ACC Testing (%)	p-value	SEN Testing (%)	SPEC Testing (%)	BAC Testing (%)	PPV Testing (%)	NPV Testing (%)	ACC Testing (%)	ACC Testing (%)
ReHo	88.65±4.11	92.26±1.84	0.001	87.64±8.34	94.71±5.25	91.17±2.38	91.14±8.21	93.18±3.94	78.15±7.05	73.84±6.75
fALFF	91.60±3.59	89.76±3.56	0.001	85.69±11.91	92.08±6.05	88.89±4.67	86.68±8.94	92.67±5.94	66.47±9.00	62.16±10.38
ALFF	85.54±4.30	90.18±4.52	0.001	81.94±11.34	94.75±4.19	88.35±5.76	89.88±7.59	90.85±5.59	76.47±8.14	83.73±8.00
Degree Centrality	97.67±2.75	90.09±2.05	0.001	81.39±8.96	94.71±4.20	88.05±3.18	90.32±7.06	90.72±3.63	74.28±7.98	78.19±8.66
LeftHC-based rsFC	93.09±3.37	90.09±2.90	0.001	86.53±6.65	92.00±6.13	89.26±2.41	86.58±8.89	92.92±3.29	64.38±7.78	64.78±11.60
RightHC-based rsFC	91.09±3.86	85.72±3.52	0.001	71.25±3.24	93.38±4.45	82.31±5.48	86.15±8.02	86.46±5.88	59.24±9.63	59.66±7.35
LeftPCC-based rsFC	94.37±3.62	87.68±2.28	0.001	80.00±2.20	92.08±5.17	86.04±4.07	86.20±8.19	89.64±5.75	65.60±6.22	62.63±7.26
RightPCC-based rsFC	94.17±2.39	85.72±2.85	0.001	81.53±6.14	88.13±5.96	84.83±2.84	80.63±7.21	89.41±2.86	59.20±8.79	58.75±11.31
LeftPCu-based rsFC	95.89±3.03	89.38±2.11	0.001	84.44±7.93	92.13±4.12	88.28±2.86	86.10±6.21	91.73±4.05	69.87±11.95	69.46±10.11
RightPCu-based rsFC	93.67±2.03	90.49±2.68	0.001	84.03±10.06	94.00±4.92	89.01±3.76	89.49±8.06	91.92±4.98	71.25±9.35	69.11±12.56
**Concatenation**	**95.50**±**4.88**	**94.45**±**2.06**	**0.001**	**83.67**±**8.78**	**96.67**±**5.67**	**90.17**±**2.59**	**95.67**±**9.02**	**91.07**±**3.90**	**75.24**±**1.13**	**80.34**±**7.26**

**Table 6:** 10-fold cross-validation mean classification performance for MCI against CN of multi-functional features at p-value = 0.05 with In-house cohort.

Feature Measure	ELM								SVM-RBF	SVM-Linear
	ACC Training (%)	ACC Testing (%)	p-value	SEN Testing (%)	SPEC Testing (%)	BAC Testing (%)	PPV Testing (%)	NPV Testing (%)	ACC Testing (%)	ACC Testing (%)
ReHo	82.10±1.66	81.76±2.69	0.001	75.38±2.43	87.58±7.99	81.48±2.89	85.84±6.78	81.30±5.77	60.18±4.58	60.89±4.97
fALFF	82.59±3.80	78.19±3.04	0.001	75.71±2.23	80.21±1.95	77.96±3.04	78.30±3.79	80.70±2.68	58.49±3.85	60.96±6.22
ALFF	87.51±3.87	84.51±2.98	0.001	81.92±3.68	86.83±4.35	84.38±3.18	84.81±4.85	85.22±6.27	74.70±2.08	77.14±7.79
Degree Centrality	81.11±2.42	82.41±3.15	0.001	77.25±3.69	86.88±5.96	82.06±2.96	84.10±5.97	81.51±2.34	67.62±3.69	68.66±3.06
LeftHC-based rsFC	76.61±1.86	78.87±4.70	0.001	78.68±3.59	78.92±4.67	78.80±4.80	76.90±6.67	81.68±2.36	62.66±6.39	63.71±6.39
RightHC-based rsFC	79.15±2.10	78.55±4.35	0.001	75.93±1.24	80.96±4.90	78.45±4.42	78.73±6.68	80.34±4.48	58.84±3.85	61.64±3.95
LeftPCC-based rsFC	79.07±1.05	81.34±4.57	0.001	75.88±2.57	86.13±5.91	81.00±6.08	82.83±5.61	81.16±7.79	67.24±4.47	68.30±3.39
RightPCC-based rsFC	78.67±1.85	79.97±4.50	0.001	75.77±1.27	83.50±5.68	79.63±4.78	80.48±4.45	80.62±4.78	63.69±5.52	63.71±4.57
LeftPCu-based rsFC	80.17±5.09	79.91±3.45	0.001	74.12±6.80	84.75±6.77	79.44±3.29	81.98±8.22	79.33±3.24	60.92±6.67	61.61±6.49
RightPCu-based rsFC	78.76±1.51	81.37±3.93	0.001	78.85±5.69	83.54±6.47	81.19±3.87	81.15±7.06	82.09±4.11	66.56±5.70	69.36±6.48
**Concatenation**	**86.52**±**6.99**	**87.20**±**2.35**	**0.001**	**78.85**±**6.90**	**87.50**±**5.72**	**81.69**±**2.75**	**84.66**±**5.12**	**81.27**±**5.70**	**69.74**±6.81	**71.13**±4.71

### Classification results: Group differences and classifications

To date, there are no guidelines available for the optimal user-defined threshold of significance (p-values) to select the relevant features to be used in machine learning for the differentiation of AD and MCI vs. CN [5, 44]. To investigate the effects of univariate statistical p-values, we show in Table 7 the ELM classification performance at different p-values (p = 0.05, 001 and 0.001). Interestingly, the best performance was found with the least significant difference (p-value = 0.05) for both datasets and both classification problems (AD vs. CN and MCI vs. CN). Specifically, in the ADNI2 cohort, the maximal mean accuracy in AD vs. CN classification was 89.92% (p-value<0.001), with a sensitivity of 86.51%, specificity of 84.17%; while in classifying MCI vs. CN, the maximal accuracy was 85.81% (p-value<0.001), with a sensitivity of 86.67%, and a specificity of 85.83%. For the in-house cohort, we achieved, in AD vs. CN classification, a maximal accuracy of 94.45% (p-value<0.001), a sensitivity of 83.67%, a specificity of 96.67%; while for the MCI vs. CN classification the maximal accuracy was 87.20% (p-value<0.001), with a sensitivity of 78.85%, and a specificity of 87.50%. Therefore we can conclude that a highly significant group difference (p-value = 0.01, 0.001) does not necessarily result in a stronger classification performance, and, conversely, that a high classification performance does not necessarily mean that strong differences exist between the means of the groups.

**Table 7:** The effects of significant p-values on the classification performances reported with ADNI2 and in-house cohorts.

**ADNI2 cohort**							
**Measure**	**p-values**	**AD *against* CN**			**MCI *against* CN**		
		**ACC (%) Testing**	**SEN (%)**	**SPEC (%)**	**ACC (%) Testing**	**SEN (%)**	**SPEC (%)**
Concatenation	**0.05**	**89.92**±**1.23**	**86.51**±**6.10**	**84.17**±**6.87**	**85.81**±**3.53**	**86.67**±**2.71**	**85.83**±**6.69**
	0.01	84.29±3.25	78.33±3.01	90.00±2.84	83.24±4.94	87.50±3.27	80.83±5.09
	0.001	85.68±3.89	85.00±4.12	85.17±4.10	83.81±3.47	91.67±4.69	73.80±6.27

**In-house cohort**							
**Measure**	**p-values**	**AD *against* CN**			**MCI *against* CN**		
		**ACC (%) Testing**	**SEN (%)**	**SPEC (%)**	**ACC (%) Testing**	**SEN (%)**	**SPEC (%)**
Concatenation	**0.05**	**94.45**±**2.06**	**83.67**±**8.78**	**96.67**±**5.67**	**87.20**±**2.35**	**78.85**±**6.90**	**87.50**±**5.72**
	0.01	91.00±2.79	84.03±6.47	94.67±5.61	84.16±4.82	78.79±5.83	88.75±5.49
	0.001	91.87±3.57	84.92±4.06	94.04±3.79	84.03±3.94	78.50±6.47	88.43±6.02

### Classification results: Hybrid combination of MVPA methods

In the previous section, we reported the results using only univariate t-tests, not combined with MVPA methods, for discriminating AD and MCI from CN. In this section we will examine the hybrid combinations of t-tests and multivariate techniques, including LASSO and SVM-RFE. Table 8 presents the performance in AD and MCI discrimination using the ELM classifier with only the univariate t-test (on concatenated features), and its combination with LASSO or SVM-RFE. The results show that the ELM classifier combined with the hybrid feature optimization framework outperformed the same classifier without feature optimization, in both cohorts and in both AD and MCI discrimination (accuracies up to 98.86% for AD and 98.57% for MCI diagnosis in the ADNI2 cohort; up to 98.70% for AD and 94.16% for MCI diagnosis in the in-house cohort). In addition, the ELM performance with combined univariate t-test and SVM-RFE is clearly superior to that of combined univariate t-test and LASSO. Interestingly, the hybrid combinations of univariate t-test with different threshold p-values and SVM-RFE resulted in similar accuracies. These similar performances can be explained as follows: In this paper, we chose the highest ranked features using grid search cross validation method on only training data, and SVM-RFE eliminated the remaining, low-ranked features. Even though with different p-values, the number of highest features are the same for the classifiers, and that resulted in equal performance.

**Table 8:** The effects of multivariate feature optimization methods (LASSO and SVM-RFE) on the ELM classification performances reported with ADNI2 and in-house cohorts.

ADNI2 cohort							
Measure	Feature optimization methods	AD *against* CN			MCI *against* CN		
		ACC (%) Testing	SEN (%)	SPEC (%)	ACC (%) Testing	SEN (%)	SPEC (%)
univariate t-test with p-value = 0.05	univariate t-test	89.92±1.23	86.51±6.10	84.17±6.87	85.81±3.53	86.67±2.71	85.83±6.69
	univariate t-test + LASSO	96.14±7.71	98.33±4.61	93.67±5.83	90.48±5.46	90.83±2.92	90.52±3.93
	**univariate t-test + SVM-RFE**	**98.86**±**4.52**	**100.00**±**0.00**	**97.50**±**2.91**	**98.57**±**4.52**	**100.00**±**0.00**	**97.50**±**7.91**
univariate t-test with p-value = 0.01	univariate t-test	84.29±3.25	78.33±3.01	90.00±2.84	83.24±4.94	87.50±3.27	80.83±5.09
	univariate t-test + LASSO	94.05±7.72	94.17±2.45	93.33±4.05	85.71±3.97	81.67±5.99	90.00±2.50
	**univariate t-test + SVM-RFE**	**98.86**±**4.52**	**100.00**±**0.00**	**97.50**±**2.91**	**98.57**±**4.52**	**100.00**±**0.00**	**97.50**±**7.91**
univariate t-test with p-value = 0.001	univariate t-test	85.68±3.89	85.00±4.12	85.17±4.10	83.81±3.47	91.67±4.69	73.80±6.27
	univariate t-test + LASSO	89.05±2.86	84.17±3.06	93.33±2.08	93.57±3.38	97.50±1.97	90.00±1.61
	**univariate t-test + SVM-RFE**	**98.86**±**4.52**	**100.00**±**0.00**	**97.50**±**2.91**	**98.57**±**4.52**	**100.00**±**0.00**	**97.50**±**7.91**
**In-house cohort**							
**Measure**	**Feature optimization methods**	**AD *against* CN**			**MCI *against* CN**		
		**ACC (%) Testing**	**SEN (%)**	**SPEC (%)**	**ACC (%) Testing**	**SEN (%)**	**SPEC (%)**
univariate t-test with p-value = 0.05	univariate t-test	94.45±2.06	83.67±8.78	96.67±5.67	87.20±2.35	78.85±6.90	87.50±5.72
	univariate t-test + LASSO	96.16±3.48	95.14±4.61	96.67±3.73	87.62±4.49	83.44±6.17	88.43±5.05
	**univariate t-test + SVM-RFE**	**98.70**±**2.25**	**99.50**±**7.91**	**97.33**±**3.44**	**94.16**±**5.67**	**92.69**±**3.17**	**95.63**±**4.39**
univariate t-test with p-value = 0.01	univariate t-test	91.00±2.79	84.03±6.47	94.67±5.61	84.16±4.82	78.79±5.83	88.75±5.49
	univariate t-test + LASSO	96.63±3.16	93.89±4.59	98.00±5.71	89.40±4.16	88.94±3.69	89.40±5.04
	**univariate t-test + SVM-RFE**	**98.70**±**2.25**	**99.50**±**7.91**	**97.33**±**3.44**	**94.16**±**5.67**	**92.69**±**3.17**	**95.63**±**4.39**
univariate t-test with p-value = 0.001	univariate t-test	91.87±3.57	84.92±4.06	94.04±3.79	84.03±3.94	78.50±6.47	88.43±6.02
	univariate t-test + LASSO	94.08±3.14	90.14±5.06	96.04±4.83	88.12±4.01	86.98±3.58	91.24±4.86
	**univariate t-test + SVM-RFE**	**98.70**±**2.25**	**99.50**±**7.91**	**97.33**±**3.44**	**94.16**±**5.67**	**92.69**±**3.17**	**95.63**±**4.39**

## Discussion

### Comparison with previous studies

In recent years, many studies have been carried out to classify AD/MCI subjects using rs-fMRI. Studies based on the use of a binary classification reported accuracies from about 75% to about 95% [18, 19]. Table 9 summarizes the results of recently published studies using rs-fMRI neuroimaging-based machine learning to discriminate AD and MCI from CN and compares them with our results. It should be noted that our method outperformed the ones proposed in [26, 50–52], which used the same MCI and CN subject selection from the ADNI2 cohort. Direct performance comparison with other studies would not be fair, because of the different datasets, preprocessing pipelines, feature measures, and classifiers. Nevertheless, it is noteworthy that the method we propose achieved the highest accuracy among all the methods described in the classification of AD and MCI vs. CN using only rs-fMRI data.

**Table 9:** Comparison of classification accuracy of AD/MCI subjects with state-of-the-art methods using rs-fMRI.

Modality	Disorder	Dataset	Feature Measures	Classifier	Accuracy (%)	Reference
rs-fMRI	AD	AD: 77, CN: 173	Seed-based FC, ALFF, ICA, concatenation	AUC	85	De Vos et al., 2018 [53]
rs-fMRI	AD	AD: 12, CN: 12	ROI-based difference between DMN and SN map	LDA	92	Zhou et al., 2010 [54]
rs-fMRI	AD	AD: 34, CN: 45	Graph measures	naïve Bayes	93.3	Khazaee et al., 2017 [3]
rs-fMRI	AD	AD: 15, CN: 16	Averaged voxel intensities of selected resting-state network	Multivariate ROC	95	Wu et al., 2013 [55]
rs-fMRI	AD	AD: 20, CN: 20	Graph measure based on FC analysis among ROIs	SVM	97	Khazaee et al., 2015 [56]
rs-fMRI	AD	AD: 27, CN: 39	FC among selected AAL regions	Bayesian Gaussian process logistic regression	97	Challis et al., 2015 [57]
**rs-fMRI**	**AD**	**AD: 33, CN: 31 (ADNI2) / AD: 81, CN: 152 (in-house)**	**Proposed methods**	**ELM**	**98.86 (ADNI2) / 98.70 (in-house)**	

rs-fMRI	MCI	*MCI: 31, CN: 31	HMM+SDL	SVM	62.90	Eavani et al., 2013 [50, 52]
rs-fMRI	MCI	*MCI: 31, CN: 31	gSR	SVM	66.13	Wee et al., 2014 [26,52]
rs-fMRI	MCI	*MCI: 31, CN: 31	sDFN	SVM	70.97	Leonardi et al., 2013 [51, 52]
rs-fMRI	MCI	*MCI: 31, CN: 31	DAE+HMM	SVM	72.58	Suk et al., 2016 [52]
rs-fMRI	MCI	MCI: 50, CN: 39	FC among selected AAL regions	Bayesian Gaussian process logistic regression	81	Challis et al., 2015 [57]
rs-fMRI	MCI	MCI: 12, CN: 25	Local connectivity and global topological properties	Multiple kernel learning	91.9	Jie et al., 2014 [58]
rs-fMRI	MCI	MCI: 89, CN: 45	Graph measures	naïve Bayes	93.3	Khazaee et al., 2017 [3]
rs-fMRI	MCI	MCI: 29, CN: 21	N/A	N/A	95.6	Beltrachini et al., 2015 [59]
**rs-fMRI**	**MCI**	*MCI: 31, CN: 31 (ADNI2) / MCI: 132, CN: 152 (in-house)	**Proposed methods**	**ELM**	**98.57 (ADNI2) / 94.16 (in-house)**	

Abbreviation: ICA = independent component analysis, AUC = area under the curve, DMN = default mode network, SN = salience network, LDA = linear discriminant analysis, ROC = receiver operating characteristic, ROI = region of interest, AAL = automated anatomical labeling, HMM = hidden markov model, SDL = sparse dictionary learning, gSR = group sparse representation, sDFN = sliding window-based dynamic functional network, DAE = deep auto encoder.

^*^ indicates the studies that used the same dataset.

### Feature selection techniques on ADNI cohort

Recent years have shown wide applications of MVPA feature selection methods applied on neuroimaging data sets from public ADNI cohorts. In Table 10, we summarize the results of previous works that applied univariate and MVPA as well as their hybrid combinations for discriminating the AD and MCI patients. In recent study [60], Kim et al. proposed multi-model hierarchical ELM integrated with t-test and LASSO applied on ROI-based features for classifications of AD and MCI against CN. Volume and mean intensity extracted from 93 ROIs of preprocessed MRI and FDG-PET images, respectively, as well as CSF values were used as features. The maximal accuracies achieved by t-test method were 96.11% and 86.15% while LASSO-based method achieved 96.03% and 86.17% for AD and MCI vs. CN, respectively. Similar AD/MCI identification framework [61] used multiple-kernel SVM method to combine the biomarkers of three modalities (MRI, FDG-PET, and CSF). Simple feature selection based on t-test was implemented, leading to the highest classification accuracies of 93.2% and 76.4% for respective AD and MCI diagnosis compared to using all features. Another study [62] used LASSO-based feature selection on GM and WM volume maps to achieve maximal accuracies of 85.7% and 81.1%. Hidalgo-Muñoz et al. [63] compared voxel-wise feature selections, i.e univariate t-test and multivariate SVM-RFE for classification of AD patients from CN using segmented GM and WM maps. Their obtained results have suggested that SVM-RFE selects discriminant features more efficiently than t-test significance for classification purposes (99.7% vs. 93.2%). Using rs-fMRI data, Khazaee et al. [56] computed functional brain network-based features, and used univariate Fisher score for feature selection and SVM as the classifier for AD classification, achieving up to a maximum accuracy of 97%. More recently, other MVPA techniques, such as principal component analysis (PCA) and independent component analysis (ICA), have been developed to keep informative features while disregarding uninformative sources of noises. Salvatore et al. used PCA method to reduce the dimensions of WM and GM density maps [64]. The reduced density maps were used for SVM classifiers to identify AD (accuracy = 76%) and MCI (accuracy = 72%) patients from CN. Similar predictive improvements due to a single MVPA feature selection or their hybrid combinations were obtained in unimodal rs-fMRI studies [26, 27], sMRI [60, 63, 65], PET [60, 66] and multi-model sMRI+PET [60, 66].

**Table 10:** Comparison of classification performances of AD/MCI patients on ADNI cohort with hybrid MVPA feature selections.

Reference	Modality	Dataset AD/MCI/CN	Feature Measures	Classifier	Feature selection	Accuracy (%)	
						AD vs. CN	MCI vs. CN
Salvatore et al., 2015 [64]	sMRI	137/76/162	sMRI: WM and GM density maps	SVM	PCA	76	72
Chu et al., 2011 [70]	sMRI	131/261/188	sMRI: voxel-wise GM	SVM	t-test	82.0	66.0
					t-test+ROI	85.0	68.0
					SVM-RFE	84.0	67.0
					t-test+SVM-RFE	85.0	67.0
Casanova et al., 2011 [62]	sMRI	49/-/49	sMRI: voxel-wise GM and WM volume maps		LASSO	85.7	-
Retico et al., 2015 [65]	sMRI	200/400/200	sMRI: voxel-wise GM maps	SVM	whole-brain; t-test; t-test+SVM-RFE	88.9 (AUC)	70.7 (AUC)
Dai et al., 2012 [31]	sMRI (OASIS data)	39/-/44	sMRI: cortical thickness	SVM	t-test+LLB; Corr+LLB; BSSWSS+LLB; t-test+RFE; Corr+RFE; BSSWSS+RFE	90.4	-
Wee et al., 2013 [27]	sMRI	198/200/200	sMRI: correlation of regional mean cortical thickness	SVM	t-test+mRMR +SVM-RFE	92.35	83.75
Zhang et al., 2011 [61]	sMRI+PET-CSF	51/99/52	sMRI: Volumetric features from sMRI and PET	SVM	t-test	93.2	76.4
Hidalgo-Muñoz et al., 2014 [63]	sMRI	185/-/185	sMRI: voxel-based GM and WM	SVM	t-test	93.2	-
					SVM-RFE	99.7	-
Wee et al., 2013 [26]	rs-fMRI	-/25/25	rs-fMRI: Pearson correlation-based FC	SVM	t-test+mRMR +SVM-RFE	-	84.0
Jie et al., 2014 [28]	fMRI (non ADNI)	-/12/25	fMRI: topological similarity between connectivity networks	SVM	t-test+RFE	-	91.9
Kim et al., 2018 [60]	sMRI+FDG-PET	51/99/52	sMRI: 93 ROI GM volume FDG-PET: 93 ROI mean intensity	ELM	t-test	96.11	86.15
					LASSO	96.03	86.17
Beheshti et al., 2016 [67, 68]	sMRI	160/-/162	sMRI: voxel-wise GM	SVM	LASSO	88.70	-
					t-test+Fisher criterion	93.01	-
Lopez et al., 2010 [69]	PET	53/114/52	Voxel-wise intensity	SVM ANN	PCA+LDA; PCA+FDR	96.7	82.19
	SPECT (non ADNI)	50/-/41				89.5	-
Khazaee et al., 2015 [56]	rs-fMRI	20/-/20	Graph measure based on FC analysis among ROIs	SVM	Fisher score	97	-
**Proposed method**	**fMRI**	**34/31/31**	**fMRI: voxel-wise regional spontaneous and functional connectivity measures**	**ELM** **SVM**	**t-test+ SVM-RFE**	**98.86**	**98.57**
		**81/132/152** **(non ADNI)**			**t-test+LASSO**	**98.70**	**94.16**

Abbreviation: PCA = principal component analysis, AUC = area under the curve, GM = gray matter, WM = white matter, mRMR = minimum redundancy and maximum relevance, LDA = linear discriminant analysis, LLB = local-learning-based feature selection, BSSWSS = between-group sum of squares (BSS) to within group sum of squares (WSS), Corr = correlation, FDR = Fisher discriminant ratio, ANN = artificial neural network.

The hybrid combinations of feature selection methods were demonstrated to diagnose the AD and MCI diseases with success. In studies [26, 27], Wee et al. combined two filter-based methods (t-test and minimum redundancy and maximum relevance-mRMR) and wrapper-based SVM-RFE methods to select the most discriminative functional connectivity extracted from rs-fMRI images. They reported maximum accuracies of 92.35% and 84% for identifications of AD and MCI patients from healthy controls. In other studies [67, 68], a new hybrid voxel-wise feature selection approach that combines t-test with Fisher criterion-based genetic algorithm was proposed predict AD patients from CNs using segmented GM images. They reported that the hybrid method’s performance (accuracy: 93.01%) is superior to those with PCA-based feature selection method (88.70%) and with no feature selection (accuracy: 87.63%). In addition, combinations of PCA with LDA and FDR (Fisher discriminant ratio) as feature selection methods outperform the whole-brain vovel-wise approach as they achieved AD classification accuracy results of up to 96.7% and 89.5% for PET and SPECT images, respectively [69].

By contrast, some studies have reported that feature selection without utilizing prior knowledge did not increase classification accuracy. Chu et al. [70] compared four common feature selection methods: 1) pre-selected ROIs based on pre-knowledge, 2) univariate t-test, 3) RFE, and 4) t-test constrained by ROIs, extracted from segmented GM maps from T1 MRI scans of three patient groups (AD, MCI, CN). Surprisingly, the results showed that: 1) the predictive accuracies with either univariate t-test or RFE were no better than those achieved using the whole brain data, 2) the hybrid method (t-test + ROI) that used the ROI as spatially constrain and t-test as the ranking of features did show significant improvements of classification accuracy in AD vs. CN and MCI vs. CN. Similarly, voxel-wise hybrid combinations of t-test and SVM-RFE applied to whole-brain GM maps were not significantly improved the AD- and MCI-diagnosis performances as compared to whole-GM approach [65].

Hybrid combinations of feature selection methods have also been used for AD and MCI classifications using other cohorts rather than standard ADNI data sets. Typically, Jie et al. [28] combined t-test and RFE to select the most topological features extracted from fMRI scans for MCI discrimination from CN subjects. They reported a maximal accuracy of 91.9%. Other study [31] utilized a hybrid feature selection approach that combines three filter- and two wrapper-based methods, and compared the performance of six different combinations of them. They reported the best accuracy of 90.4% using the proposed hybrid approach with SVM classifier in LOO-CV for AD patients diagnosis taken from Open Access Series of Imaging Studies (OASIS) database (http://www.oasis-brains.org/).

### The benefits of MVPA feature reduction methods

It is known that the performance of pattern recognition methods such as SVM and ELM decreases with the increase of non-informative features [19]. Machine learning techniques take advantage of the multivariate nature of the fMRI data and are able to identify maximally discriminative spatial patterns [58]. In the present work, we have examined and assessed an approach for fMRI pattern discrimination analysis based on ELM and hybrid combinations of multi-voxels, including univariate and MVPA feature reductions. Our results show that the conventional univariate t-test, as used alone, can be used with a classifier for identification of AD/MCI patients. In addition, as shown in Table 7, a very low p-value cut-off does not guarantee a strongly informative feature, while a larger p-value does not necessarily indicate an irrelevant feature. Thus, by discarding voxels based only on the results of statistical tests sensitive to group means, could lead to loss of discriminative ability. Therefore, additional MVPA methods should be used in combination with the univariate group-level t-test.

We also demonstrated that the hybrid combination of multi-voxel methods (t-test + SVM-RFE and t-test + LASSO) increases the discriminative power of the patterns (Table 8). In our studies, we searched for the most relevant discriminative patterns using SVM-RFE, which iteratively eliminates the lowest-ranked patterns based on multivariate information classified by RBF-based SVM; and LASSO, which chooses the sparse features that contribute the most to the accuracy of the model during training. It is worth noting that because of the lesser sensitivity of the univariate method, the wisest setting for combining univariate and multivariate is to use larger p-value thresholds (thus preventing the exclusion of potentially relevant voxels), and then remove irrelevant voxels based on multivariate ranking functions.

### Clinical significance of the results

The regions showing significant changes in a univariate t-test play an important role in achieving highly accurate differential diagnosis when used in combination with MVPA feature reduction methods. The following discussion of the significant regions may have clinical relevance.

We showed that the highest discrimination patterns were achieved when all information from regional coherence and functional connectivity measures were combined. This may imply that different parts of the brain undergo different functional failures as a consequence of AD/MCI. Therefore, classification methods should include the maximum amount of informative change to achieve optimal discrimination.

**Fig 5:**
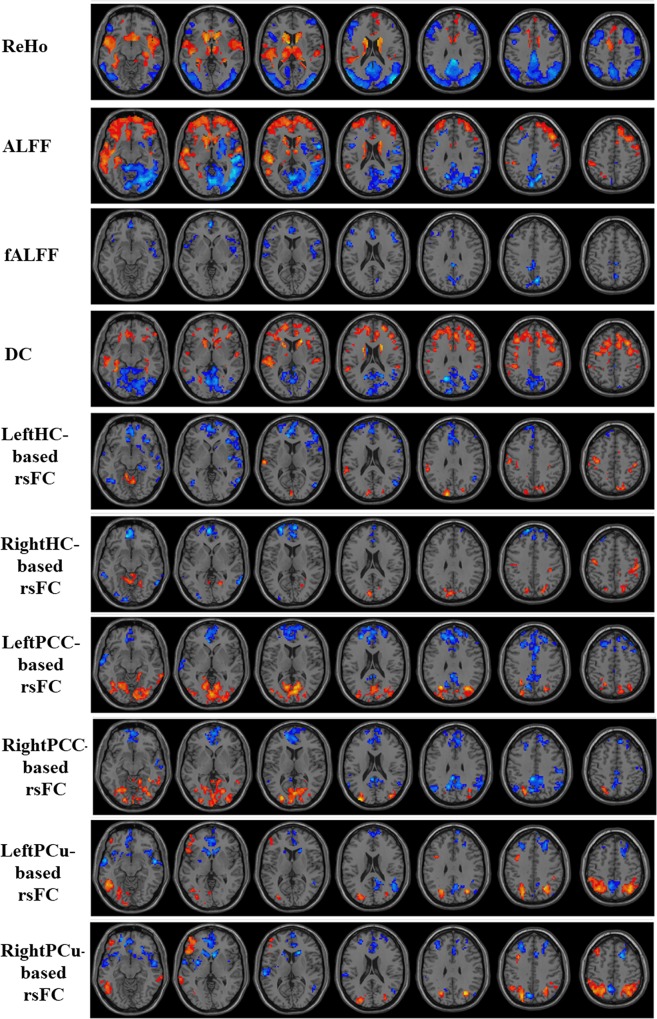
Univariate t-statistical difference maps between AD and CN groups of ten measures extracted from in-house cohort. Voxels with p-value<0.05 and cluster size of 85 voxels (2295 mm^3^) corresponding to a corrected p-value<0.05 were used to identify the significant clusters. Hot and cold colours indicate AD-related measures increases and decreases, respectively.

**Fig 6:**
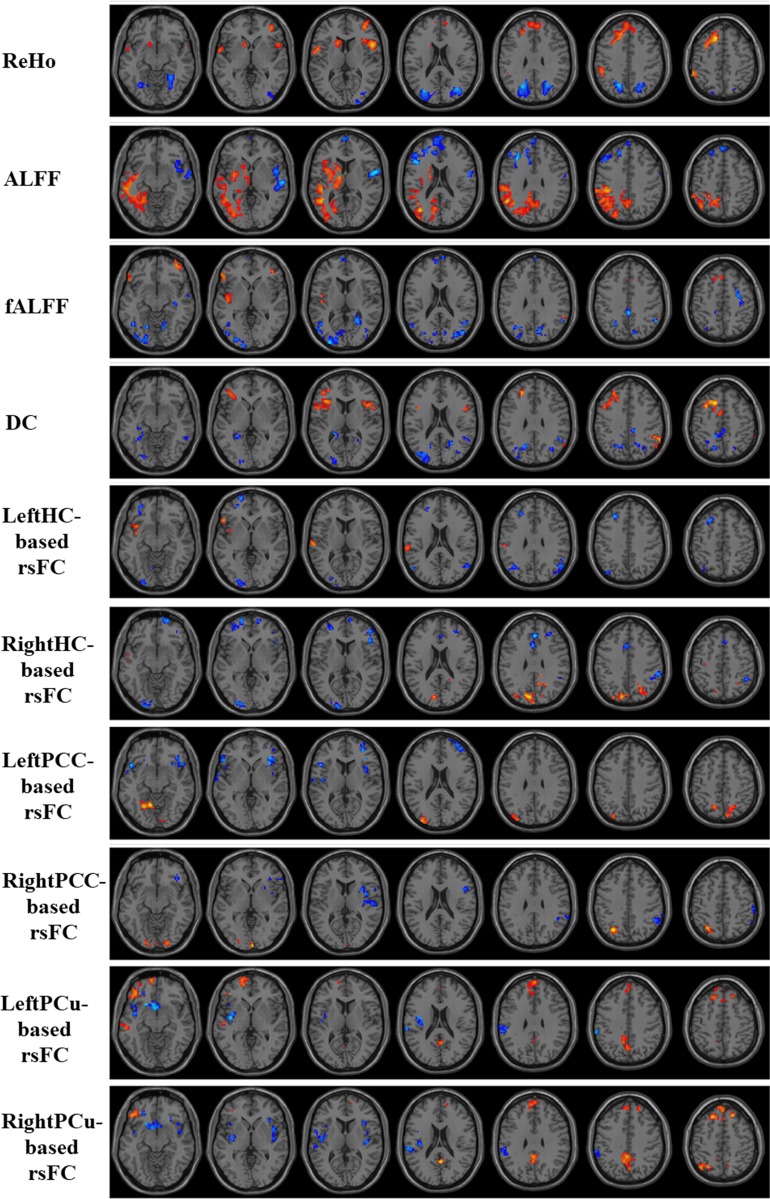
Univariate t-statistical difference maps between MCI and CN groups of ten measures extracted from in-house cohort. Voxels with p-value<0.05 and cluster size of 85 voxels (2295 mm^3^) corresponding to a corrected p-value<0.05 were used to identify the significant clusters. Hot and cold colours indicate MCI-related measures increases and decreases, respectively.

One important finding of the current study is that the significant regional features depend on the dataset: Therefore we cannot label any regional feature as a global biomarker of AD or MCI. Our binary classification results between folds indicated that the significant features are subject to change when the cross-validation subgroups of AD and MCI subjects are changed. Therefore, no specific regional feature would be an appropriate global biomarker for AD and MCI diagnosis. For instance, Figs 5 and 6 present an example of the statistical group-level differences between AD and CN, and between MCI and CN, for all measures of a CV fold. Regions with significant changes were mostly located in the DMN (mainly involving in the prefrontal cortex, the PCu, and the PCC).

### Limitations and future perspectives

Notwithstanding the discriminative power of the framework we presented for AD and MCI, this work has several limitations that we now describe. First, the limited sample size of the in-house cohort (81 AD, 132 MCI, and 152 CN), but especially of the ADNI2 one (33 AD, 31 MCI, and 31 CN), prevented the algorithm from learning during the training phase. Therefore these small datasets certainly do not adequately represent the patient population, so that the generalization of our results to other groups is not guaranteed.

A second limitation has to do with model complexity, as our proposed voxel-wise method may require more computation and resources than methods based on regions-of-interest (ROIs). However, the computation and resource burden only occur in the training phase, which can be implemented offline, whereas the computation for testing consists of simple functions. Thus, from the clinical perspective, we believe such limitation is acceptable when considering the better accuracies obtained.

Third, our multi-measure classification framework only considers functional MRI data. However, it is expected that combining as many modalities as possible would be advantageous for the discrimination of AD and MCI from CN [71]. Accordingly, in future studies, we plan to develop a multi-modal classification framework combining multiple data sources, including structural MRI and PET data.

## Conclusion

In conclusion, we proved the possibility of using rs-fMRI scans for AD/MCI prediction in individual subjects. Using a standard Alzheimer’s disease Neuroimaging Initiative cohort and an in-house AD cohort from South Korea, the proposed framework extracts the maximum amount of information changes due to AD/MCI from concatenations of multiple rs-fMRI biomarkers which lead to maximal classification accuracies as compared to all other recent researches. The combination of t-test-based univariate, and RFE-based multivariate feature selection techniques performed on the concatenated measure extracted from rs-fMRI data provided the best discriminative performance when the features thus selected were used by the ELM classifier, superior to that of linear and non-linear SVM classifiers. These results may direct future studies using rs-fMRI scans for the classification of patients with preclinical AD or MCI.

## Supporting information

S1 Table The subject IDs of three groups of ADNI2 cohort used in this study. (DOC)

## References

[1] Braak H, Braak E. Neuropathological stageing of Alzheimer-related changes. Acta Neuropathol. 1991;82: 239–259. 1759558 10.1007/BF00308809

[2] Zheng W, Yao Z, Xie Y, Fan J, Hu B. Identification of Alzheimer’s Disease and Mild Cognitive Impairment Using Networks Constructed Based on Multiple Morphological Brain Features. Biological Psychiatry: Cognitive Neuroscience and Neuroimaging. 2018; 10.1016/j.bpsc.2018.06.004 30077576

[3] Khazaee A, Ebrahimzadeh A, Babajani-Feremi A. Classification of patients with MCI and AD from healthy controls using directed graph measures of resting-state fMRI. Behav Brain Res. 2017;322: 339–350. 10.1016/j.bbr.2016.06.043 27345822

[4] Mahmoudi A, Takerkart S, Regragui F, Boussaoud D, Brovelli A. Multivoxel Pattern Analysis for fMRI Data: A Review. Comput Math Methods Med. 2012;2012: 1–14. 10.1155/2012/961257 PMC3529504 23401720

[5] Mwangi B, Tian TS, Soares JC. A Review of Feature Reduction Techniques in Neuroimaging. Neuroinformatics. 2013;12: 229–244. 10.1007/s12021-013-9204-3 PMC4040248 24013948

[6] Ashburner J, Friston KJ. Voxel-Based Morphometry—The Methods. Neuroimage. 2000;11: 805–821. 10.1006/nimg.2000.0582 10860804

[7] Yang Z, Fang F, Weng X. Recent developments in multivariate pattern analysis for functional MRI. Neurosci Bull. 2012 10.1007/s12264-012-1253-3 22833038 PMC5561894

[8] Chaves R, Ramírez J, Górriz JM, López M, Salas-Gonzalez D, Alvarez I, et al SVM-based computer-aided diagnosis of the Alzheimer’s disease using t-test NMSE feature selection with feature correlation weighting. Neurosci Lett. 2009;461: 293–297. 10.1016/j.neulet.2009.06.052 19549559

[9] Zang Y, Jiang T, Lu Y, He Y, Tian L. Regional homogeneity approach to fMRI data analysis. Neuroimage. 2004;22: 394–400. 10.1016/j.neuroimage.2003.12.030 15110032

[10] He Y, Wang L, Zang Y, Tian L, Zhang X, Li K, et al Regional coherence changes in the early stages of Alzheimer’s disease: a combined structural and resting-state functional MRI study. Neuroimage. 2007;35: 488–500. 10.1016/j.neuroimage.2006.11.042 17254803

[11] Zou Q-H, Zhu C-Z, Yang Y, Zuo X-N, Long X-Y, Cao Q-J, et al An improved approach to detection of amplitude of low-frequency fluctuation (ALFF) for resting-state fMRI: fractional ALFF. J Neurosci Methods. 2008;172: 137–141. 10.1016/j.jneumeth.2008.04.012 18501969 PMC3902859

[12] Guo Z, Liu X, Li J, Wei F, Hou H, Chen X, et al Fractional amplitude of low-frequency fluctuations is disrupted in Alzheimer’s disease with depression. Clin Neurophysiol. 2017;128: 1344–1349. 10.1016/j.clinph.2017.05.003 28570868

[13] Li Y, Jing B, Liu H, Li Y, Gao X, Li Y, et al Frequency-Dependent Changes in the Amplitude of Low-Frequency Fluctuations in Mild Cognitive Impairment with Mild Depression. J Alzheimers Dis. 2017;58: 1175–1187. 10.3233/JAD-161282 28550250

[14] Lin Q, Rosenberg MD, Yoo K, Hsu TW, O’Connell TP, Chun MM. Resting-State Functional Connectivity Predicts Cognitive Impairment Related to Alzheimer’s Disease. Front Aging Neurosci. 2018;10: 94 10.3389/fnagi.2018.00094 29706883 PMC5908906

[15] Han Y, Wang J, Zhao Z, Min B, Lu J, Li K, et al Frequency-dependent changes in the amplitude of low-frequency fluctuations in amnestic mild cognitive impairment: a resting-state fMRI study. Neuroimage. 2011;55: 287–295. 10.1016/j.neuroimage.2010.11.059 21118724

[16] Li Y, Wang X, Li Y, Sun Y, Sheng C, Li H, et al Abnormal Resting-State Functional Connectivity Strength in Mild Cognitive Impairment and Its Conversion to Alzheimer’s Disease. Neural Plast. 2016;2016: 4680972 10.1155/2016/4680972 26843991 PMC4710946

[17] Dai Z, Yan C, Li K, Wang Z, Wang J, Cao M, et al Identifying and Mapping Connectivity Patterns of Brain Network Hubs in Alzheimer’s Disease. Cereb Cortex. 2015;25: 3723–3742. 10.1093/cercor/bhu246 25331602

[18] Rathore S, Habes M, Iftikhar MA, Shacklett A, Davatzikos C. A review on neuroimaging-based classification studies and associated feature extraction methods for Alzheimer’s disease and its prodromal stages. Neuroimage. 2017;155: 530–548. 10.1016/j.neuroimage.2017.03.057 28414186 PMC5511557

[19] Arbabshirani MR, Plis S, Sui J, Calhoun VD. Single subject prediction of brain disorders in neuroimaging: Promises and pitfalls. Neuroimage. 2017;145: 137–165. 10.1016/j.neuroimage.2016.02.079 27012503 PMC5031516

[20] Guyon I, Weston J, Barnhill S, Vapnik V. Gene Selection for Cancer Classification using Support Vector Machines. Mach Learn. Kluwer Academic Publishers; 2002;46: 389–422.

[21] De Martino F, Valente G, Staeren N, Ashburner J, Goebel R, Formisano E. Combining multivariate voxel selection and support vector machines for mapping and classification of fMRI spatial patterns. Neuroimage. 2008;43: 44–58. 10.1016/j.neuroimage.2008.06.037 18672070

[22] Mishra S, Mishra D. SVM-BT-RFE: An improved gene selection framework using Bayesian T-test embedded in support vector machine (recursive feature elimination) algorithm. Karbala International Journal of Modern Science, 2015 10.1016/j.kijoms.2015.10.002

[23] Qureshi M. N. I., Min B., Jo H. J., Lee B. (2016). Multiclass classification for the differential diagnosis on the adhd subtypes using recursive feature elimination and hierarchical extreme learning machine: structural MRI Study. PLoS ONE 11:e0160697 10.1371/journal.pone.0160697 27500640 PMC4976974

[24] Qureshi M. N. I., Oh J., Min B., Jo H. J., Lee B. (2017b). Multi-modal, multi-measure, and multi-class discrimination of ADHD with hierarchical feature extraction and extreme learning machine using structural and functional brain MRI. Front. Hum. Neurosci. 11:157 10.3389/fnhum.2017.00157 28420972 PMC5378777

[25] Dai D, Wang J, Hua J, He H. Classification of ADHD children through multimodal magnetic resonance imaging. Front Syst Neurosci. 2012 9 3;6:63 10.3389/fnsys.2012.00063 22969710 PMC3432508

[26] Wee CY, Yap PT, Zhang D, Wang L, Shen D. Group-constrained sparse fMRI connectivity modeling for mild cognitive impairment identification. Brain Struct Funct. 2014 10.1007/s00429-013-0524-8 23468090 PMC3710527

[27] Wee CY, Yap PT, Shen D; Alzheimer's Disease Neuroimaging Initiative. Prediction of Alzheimer's disease and mild cognitive impairment using cortical morphological patterns. Hum Brain Mapp. 2013 12;34(12):3411–25. 10.1002/hbm.22156 22927119 PMC3511623

[28] Jie B, Zhang D, Wee CY, Shen D. Topological graph kernel on multiple thresholded functional connectivity networks for mild cognitive impairment classification. Hum Brain Mapp. 2014 7;35(7):2876–97. 10.1002/hbm.22353 24038749 PMC4116356

[29] Wee CY, Wang L, Shi F, Yap PT, Shen D. Diagnosis of autism spectrum disorders using regional and interregional morphological features. Hum Brain Mapp. 2014 7;35(7):3414–30. 25050428 10.1002/hbm.22411 PMC4109659

[30] Falahati F, Westman E, Simmons A. Multivariate data analysis and machine learning in Alzheimer's disease with a focus on structural magnetic resonance imaging. J Alzheimers Dis. 2014 10.3233/JAD-131928 24718104

[31] Dai D, Huiguang H, Joshua TV, Zengguang H. Accurate prediction of AD patients using cortical thickness networks. Machine Vision and Applications (2013). 10.1007/s00138-012-0462-0

[32] Li X, Peng S, Chen J, Lü B, Zhang H, Lai M. SVM-T-RFE: a novel gene selection algorithm for identifying metastasis-related genes in colorectal cancer using gene expression profiles. Biochem Biophys Res Commun. 2012 10.1016/j.bbrc.2012.01.087 22306013

[33] Chao-Gan Y, Yu-Feng Z. DPARSF: A MATLAB Toolbox for “Pipeline” Data Analysis of Resting-State fMRI. Front Syst Neurosci. 2010;4: 13 10.3389/fnsys.2010.00013 20577591 PMC2889691

[34] Ashburner J. A fast diffeomorphic image registration algorithm. Neuroimage. 2007;38: 95–113. 10.1016/j.neuroimage.2007.07.007 17761438

[35] Song X-W, Dong Z-Y, Long X-Y, Li S-F, Zuo X-N, Zhu C-Z, et al REST: a toolkit for resting-state functional magnetic resonance imaging data processing. PLoS One. 2011;6: e25031 10.1371/journal.pone.0025031 21949842 PMC3176805

[36] Kendall M, Gibbons JDR. Correlation methods Oxford: Oxford University Press; 1990.

[37] Zang Y, Jiang T, Lu Y, He Y, Tian L. Regional homogeneity approach to fMRI data analysis. Neuroimage. 2004;22: 394–400. 10.1016/j.neuroimage.2003.12.030 15110032

[38] Zang Y-F, He Y, Zhu C-Z, Cao Q-J, Sui M-Q, Liang M, et al Altered baseline brain activity in children with ADHD revealed by resting-state functional MRI. Brain Dev. 2007;29: 83–91. 10.1016/j.braindev.2006.07.002 16919409

[39] Zhou Y, Wang Y, Rao L-L, Liang Z-Y, Chen X-P, Zheng D, et al Disrutpted resting-state functional architecture of the brain after 45-day simulated microgravity. Front Behav Neurosci. 2014;8 10.3389/fnbeh.2014.00200 24926242 PMC4046318

[40] Zuo X-N, Ehmke R, Mennes M, Imperati D, Xavier Castellanos F, Sporns O, et al Network Centrality in the Human Functional Connectome. Cereb Cortex. 2011;22: 1862–1875. 10.1093/cercor/bhr269 21968567

[41] Zhan Z-W, Lin L-Z, Yu E-H, Xin J-W, Lin L, Lin H-L, et al Abnormal resting-state functional connectivity in posterior cingulate cortex of Parkinson’s disease with mild cognitive impairment and dementia. CNS Neurosci Ther. 2018; 10.1111/cns.12838 29500931 PMC6490031

[42] Qureshi MNI, Oh J, Cho D, Jo HJ, Lee B. Multimodal Discrimination of Schizophrenia Using Hybrid Weighted Feature Concatenation of Brain Functional Connectivity and Anatomical Features with an Extreme Learning Machine. Front Neuroinform. 2017;11: 59 10.3389/fninf.2017.00059 28943848 PMC5596100

[43] Calhoun VD, Sui J. Multimodal Fusion of Brain Imaging Data: A Key to Finding the Missing Link(s) in Complex Mental Illness. Biological Psychiatry: Cognitive Neuroscience and Neuroimaging. 2016;1: 230–244. 27347565 10.1016/j.bpsc.2015.12.005 PMC4917230

[44] Mwangi B, Ebmeier KP, Matthews K, Steele JD. Multi-centre diagnostic classification of individual structural neuroimaging scans from patients with major depressive disorder. Brain. 2012;135: 1508–1521. 10.1093/brain/aws084 22544901

[45] Wee C-Y, Yap P-T, Zhang D, Denny K, Browndyke JN, Potter GG, et al Identification of MCI individuals using structural and functional connectivity networks. Neuroimage. 2012;59: 2045–2056. 10.1016/j.neuroimage.2011.10.015 22019883 PMC3254811

[46] Yan K, Zhang D. Feature selection and analysis on correlated gas sensor data with recursive feature elimination. Sens Actuators B Chem. Elsevier; 2015;212: 353–363.

[47] Tibshirani R. Regression Shrinkage and Selection via the Lasso. J R Stat Soc Series B Stat Methodol. [Royal Statistical Society, Wiley]; 1996;58: 267–288.

[48] Wu TT, Chen YF, Hastie T, Sobel E, Lange K. Genome-wide association analysis by lasso penalized logistic regression. Bioinformatics. academic.oup.com; 2009;25: 714–721. 10.1093/bioinformatics/btp041 19176549 PMC2732298

[49] Friedman J, Hastie T, Tibshirani R. Regularization paths for generalized linear models via coordinate descent. J Stat Softw. NIH Public Access; 2010;33: 1 20808728 PMC2929880

[50] Eavani H, Satterthwaite TD, Gur RE, Gur RC, Davatzikos C. Unsupervised learning of functional network dynamics in resting state fMRI. Inf Process Med Imaging. 2013;23: 426–437. 24683988 10.1007/978-3-642-38868-2_36 PMC3974209

[51] Leonardi N, Richiardi J, Gschwind M, Simioni S, Annoni J-M, Schluep M, et al Principal components of functional connectivity: a new approach to study dynamic brain connectivity during rest. Neuroimage. 2013;83: 937–950. 10.1016/j.neuroimage.2013.07.019 23872496

[52] Suk H-I, Wee C-Y, Lee S-W, Shen D. State-space model with deep learning for functional dynamics estimation in resting-state fMRI. Neuroimage. 2016;129: 292–307. 10.1016/j.neuroimage.2016.01.005 26774612 PMC5437848

[53] de Vos F, Koini M, Schouten TM, Seiler S, van der Grond J, Lechner A, et al A comprehensive analysis of resting state fMRI measures to classify individual patients with Alzheimer’s disease. Neuroimage. 2018;167: 62–72. 10.1016/j.neuroimage.2017.11.025 29155080

[54] Zhou J, Greicius MD, Gennatas ED, Growdon ME, Jang JY, Rabinovici GD, et al Divergent network connectivity changes in behavioural variant frontotemporal dementia and Alzheimer’s disease. Brain. 2010;133: 1352–1367. 10.1093/brain/awq075 20410145 PMC2912696

[55] Wu X, Li J, Ayutyanont N, Protas H, Jagust W, Fleisher A, et al The receiver operational characteristic for binary classification with multiple indices and its application to the neuroimaging study of Alzheimer’s disease. IEEE/ACM Trans Comput Biol Bioinform. 2013;10: 173–180. 10.1109/TCBB.2012.141 23702553 PMC4085147

[56] Khazaee A, Ebrahimzadeh A, Babajani-Feremi A. Identifying patients with Alzheimer’s disease using resting-state fMRI and graph theory. Clin Neurophysiol. 2015;126: 2132–2141. 10.1016/j.clinph.2015.02.060 25907414

[57] Challis E, Hurley P, Serra L, Bozzali M, Oliver S, Cercignani M. Gaussian process classification of Alzheimer’s disease and mild cognitive impairment from resting-state fMRI. Neuroimage. 2015;112: 232–243. 10.1016/j.neuroimage.2015.02.037 25731993

[58] Jie B, Zhang D, Gao W, Wang Q, Wee C-Y, Shen D. Integration of network topological and connectivity properties for neuroimaging classification. IEEE Trans Biomed Eng. 2014;61: 576–589. 10.1109/TBME.2013.2284195 24108708 PMC4106141

[59] Beltrachini L, De Marco M, Taylor ZA, Lotjonen J, Frangi AF, Venneri A. Integration of Cognitive Tests and Resting State fMRI for the Individual Identification of Mild Cognitive Impairment. Curr Alzheimer Res. 2015;12: 592–603. 26238814 10.2174/156720501206150716120332

[60] Kim J, Lee B. Identification of Alzheimer's disease and mild cognitive impairment using multimodal sparse hierarchical extreme learning machine. Hum Brain Mapp. 2018 10.1002/hbm.24207 29736986 PMC6866602

[61] Zhang D, Wang Y, Zhou L, Yuan H, Shen D; Alzheimer's Disease Neuroimaging Initiative. Multimodal classification of Alzheimer's disease and mild cognitive impairment. Neuroimage. 2011 10.1016/j.neuroimage.2011.01.008 21236349 PMC3057360

[62] Casanova R, Whitlow CT, Wagner B, Williamson J, Shumaker SA, Maldjian JA, Espeland MA. High dimensional classification of structural MRI Alzheimer's disease data based on large scale regularization. Front Neuroinform. 2011 10.3389/fninf.2011.00022 22016732 PMC3193072

[63] Hidalgo-Muñoz AR, Ramírez J, Górriz JM, Padilla P. Regions of interest computed by SVM wrapped method for Alzheimer's disease examination from segmented MRI. Front Aging Neurosci. 2014 10.3389/fnagi.2014.00020 24634656 PMC3929832

[64] Salvatore C, Cerasa A, Battista P, Gilardi MC, Quattrone A, Castiglioni I; Alzheimer's Disease Neuroimaging Initiative. Magnetic resonance imaging biomarkers for the early diagnosis of Alzheimer's disease: a machine learning approach. Front Neurosci. 2015 10.3389/fnins.2015.00307 26388719 PMC4555016

[65] Retico A, Bosco P, Cerello P, Fiorina E, Chincarini A, Fantacci ME. Predictive Models Based on Support Vector Machines: Whole-Brain versus Regional Analysis of Structural MRI in the Alzheimer's Disease. Neuroimaging. 2015 10.1111/jon.12163 25291354 PMC4388756

[66] Ota K, Oishi N, Ito K, Fukuyama H; SEAD-J Study Group; Alzheimer's Disease Neuroimaging Initiative. Effects of imaging modalities, brain atlases and feature selection on prediction of Alzheimer's disease. J Neurosci Methods. 2015 10.1016/j.jneumeth.2015.08.020 26318777

[67] Beheshti I, Demirel H; Alzheimer’s Disease Neuroimaging Initiative. Feature-ranking-based Alzheimer's disease classification from structural MRI. Magn Reson Imaging. 2016 10.1016/j.mri.2015.11.009 26657976

[68] Beheshti I, Demirel H, Matsuda H; Alzheimer's Disease Neuroimaging Initiative. Classification of Alzheimer's disease and prediction of mild cognitive impairment-to-Alzheimer's conversion from structural magnetic resource imaging using feature ranking and a genetic algorithm. Comput Biol Med. 2017 10.1016/j.compbiomed.2017.02.011 28260614

[69] López M, Ramírez J, Górriz JM, Álvarez I, Salas-Gonzalez D, Segovia F, et al Principal component analysis-based techniques and supervised classification schemes for the early detection of Alzheimer's disease. Neurocomputing 2011 10.1016/j.neucom.2010.06.025

[70] Chu C, Hsu AL, Chou KH, Bandettini P, Lin C, Alzheimer's Disease Neuroimaging Initiative. Does feature selection improve classification accuracy? Impact of sample size and feature selection on classification using anatomical magnetic resonance images. Neuroimage. 2012 10.1016/j.neuroimage.2011.11.066 22166797

[71] Duc NT, Lee B. Microstate functional connectivity in EEG cognitive task revealed by multivariate Gaussian hidden Markov model with phase locking value. J Neural Eng 2019 10.1088/1741-2552/ab0169 30673644

