# Supplementary material for: Hybrid multivariate pattern analysis combined with extreme learning machine for Alzheimer’s dementia diagnosis using multi-measure rs-fMRI spatial patterns
Source: PLoS One. 2019 Feb 22;14(2):e0212582. doi: 10.1371/journal.pone.0212582 (PMC6386400; doi:10.1371/journal.pone.0212582)
Supplement: S1 Table — (DOC) [file pone.0212582.s001.doc]

S1 Table. The subject IDs of three groups of ADNI2 cohort used in this study.

| **Subjects** | **CN group** | **MCI group** | **AD group** |
| --- | --- | --- | --- |
| 1 | 002_S_1261 | 002_S_4237 | 002_S_5018 |
| 2 | 002_S_1280 | 002_S_4447 | 006_S_4192 |
| 3 | 002_S_4225 | 002_S_4473 | 006_S_4546 |
| 4 | 002_S_4262 | 002_S_4799 | 006_S_4867 |
| 5 | 002_S_4264 | 006_S_4679 | 010_S_5163 |
| 6 | 002_S_4270 | 012_S_4012 | 013_S_5071 |
| 7 | 006_S_4357 | 012_S_4128 | 018_S_4696 |
| 8 | 006_S_4449 | 013_S_4791 | 018_S_4733 |
| 9 | 006_S_4485 | 013_S_4917 | 018_S_5074 |
| 10 | 010_S_4442 | 018_S_2138 | 018_S_5240 |
| 11 | 013_S_4580 | 018_S_2155 | 019_S_4252 |
| 12 | 013_S_4616 | 018_S_2180 | 019_S_4477 |
| 13 | 018_S_4257 | 018_S_4597 | 019_S_4549 |
| 14 | 018_S_4349 | 018_S_4809 | 019_S_5012 |
| 15 | 018_S_4400 | 018_S_4868 | 019_S_5019 |
| 16 | 019_S_4367 | 019_S_4285 | 031_S_4024 |
| 17 | 019_S_4835 | 031_S_4005 | 053_S_5070 |
| 18 | 031_S_4021 | 031_S_4149 | 053_S_5208 |
| 19 | 031_S_4032 | 031_S_4476 | 100_S_5106 |
| 20 | 031_S_4218 | 031_S_4947 | 130_S_4589 |
| 21 | 031_S_4474 | 053_S_2357 | 130_S_4641 |
| 22 | 053_S_4578 | 053_S_2396 | 130_S_4660 |
| 23 | 100_S_4469 | 053_S_4661 | 130_S_4730 |
| 24 | 100_S_4511 | 053_S_4557 | 130_S_4971 |
| 25 | 129_S_0778 | 100_S_4556 | 130_S_4982 |
| 26 | 130_S_4343 | 129_S_4220 | 130_S_4984 |
| 27 | 130_S_4352 | 130_S_4405 | 130_S_4990 |
| 28 | 136_S_4269 | 130_S_4415 | 130_S_4997 |
| 29 | 136_S_4433 | 130_S_4417 | 130_S_5006 |
| 30 | 136_S_4726 | 130_S_4883 | 130_S_5059 |
| 31 | 136_S_4727 | 136_S_4517 | 130_S_5231 |
| 32 | - | - | 131_S_5138 |
| 33 | - | - | 136_S_4993 |
